# Proteomic Analysis of Thiol Modifications and Assessment of Structural Changes in Hemoglobin Induced by the Aniline Metabolites N-Phenylhydroxylamine and Nitrosobenzene

**DOI:** 10.1038/s41598-017-14653-w

**Published:** 2017-11-01

**Authors:** Carolina Möller, W. Clay Davis, Vanessa R. Thompson, Frank Marí, Anthony P. DeCaprio

**Affiliations:** 1 Department of Chemistry and Biochemistry, Florida International University, 11200 SW 8th St, Miami, FL 33199 USA; 2 Marine Biochemical Sciences, Hollings Marine Laboratory, National Institute of Standards and Technology, 331 Fort Johnson Rd., Charleston, SC 29412 USA

## Abstract

MS-based proteomic analysis was combined with *in silico* quantum mechanical calculations to improve understanding of protein adduction by N-phenylhydroxylamine (PhNHOH) and nitrosobenzene (NOB), metabolic products of aniline. *In vitro* adduction of model peptides containing nucleophilic sidechains (Cys, His, and Lys) and selected proteins (bovine and human hemoglobin and β-lactoglobulin-A) were characterized. Peptide studies identified the Cys thiolate as the most reactive nucleophile for these metabolites, a result consistent with *in silico* calculations of reactivity parameters. For PhNHOH, sulfinamides were identified as the primary adduction products, which were stable following tryptic digestion. Conversely, reactions with NOB yielded an additional oxidized adduct, the sulfonamide. *In vitro* exposure of human whole blood to PhNHOH and NOB demonstrated that only sulfinamides were formed. In addition to previously reported adduction of β^93^Cys of human Hb, two novel sites of adduction were found; α^104^Cys and β^112^Cys. We also report CD and UV-Vis spectroscopy studies of adducted human Hb that revealed loss of α-helical content and deoxygenation. The results provide additional understanding of the covalent interaction of aromatic amine metabolites with protein nucleophiles.

## Introduction

Covalent protein modifications (*i.e*., adducts) are usually stable entities that are expected to accumulate during chronic exposure to electrophilic xenobiotics or their metabolites. Such adducts generally have a half-life similar to the unmodified protein counterparts; consequently, they have been used as biomarkers for exposure to various environmental and occupational toxicants^1,2^. Reactive xenobiotics are frequently involved in covalent chemical modification of nucleophilic functional groups in proteins. Numerous protein adducts induced by xenobiotics of diverse origin reflecting a variety of sites and types of chemical modification have been reported^3,4^.

Important protein nucleophiles include the free thiol of cysteine, the ring nitrogens of histidine, and the ε-amine function of lysine^2^. Quantum mechanical calculations based on “Hard and Soft, Acids and Bases” (HSAB) theory are valuable for predicting the relative reactivity of electrophilic xenobiotics with selected nucleophiles^5^. As demonstrated in numerous studies, electrophiles will preferentially react with nucleophiles of similar “softness” or “hardness”, parameters that are based on the polarizability of the electron density of the reactant molecules^6,7^. HSAB parameters are also dependent on ionization state; for example, cysteine in the thiolate (anionic) form is considerably “softer” than that in the neutral thiol form. HSAB calculations have been employed to predict reactivity of electrophilic xenobiotics of various classes with protein nucleophiles^8,9^.

Human blood proteins such as hemoglobin (Hb) and serum albumin (HSA) have reactive free thiol groups and are among the most promising candidates for studying chemical adduction by potential carcinogens and other chemical hazards^10,11^. The reactivity of free thiols often resides in unusually low pKa values induced by local ionic interactions within their surrounding microenvironment, such as in the “catalytic triads” present in certain enzyme active sites^1,12,13^. Steric accessibility, mediated by tertiary and quaternary protein structure, is also an important determinant of reactivity^14,15^. In particular, the free thiols of the human Hb β-subunit (^93^Cys) and of HSA (^34^Cys), have been shown to be highly reactive to electrophilic drugs and environmental/occupational toxicants.

Aromatic amines (AAs) and heterocyclic aromatic amines (HAAs) are N-substituted aryl compounds used commercially that are also released during the combustion of tobacco, heating of cooking oil, and in meats when cooked at high temperature^16,17^. PhNHOH and NOB are reactive metabolic products of aniline, which is an aromatic amine present in some industrial processes and also in tobacco and certain foods at low levels^18^. Adverse effects of aniline, including splenic toxicity, methemoglobinemia, and hemosiderosis, have been demonstrated in animal studies. Aniline is classified by USEPA in Class B2 (*i.e*., probable human carcinogen based on animal data) based on splenic tumors in rats and by IARC in Group 3 (*i.e*., not classifiable as to its carcinogenicity to humans).

**Figure 1:**
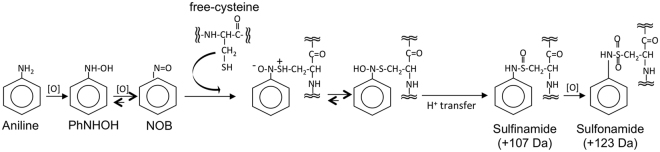
Scheme of the reaction of aniline with free thiol. First step in the reaction process consists of the N-oxidation of aniline to PhNHOH, primarily in hepatocytes. Phenylhydroxylamine is then co-oxidized to NOB, primarily in erythrocytes. NOB reacts with the SH-groups of free cysteine to form the sulfinamide, which can additionally oxidize to a sulfonamide. Expected mass increases of the reaction products are shown.

Arylamine-protein thiol adducts have been extensively studied because of their potential use as exposure biomarkers in occupational and environmental settings^19–26^. Blood protein adducts of AAs and HAAs are formed through their N-oxidized metabolites, in which the first metabolic product is the N-hydroxylated amine formed outside of the red blood cell (RBC) via a cytochrome P-450 mediated mechanism. This arylhydroxylamine is further oxidized within the RBC to form the reactive electrophilic intermediate, an arylnitroso derivative, which reacts with free thiols to form a sulfinamide protein adduct (Fig. 1)^27,28^.

Here we further explore the molecular mechanism of protein adduction by PhNHOH and NOB. The present study examined the *in vitro* reactivity of PhNHOH and NOB with various protein nucleophiles and characterized the products (*i.e*., sulfinamides, sulfonamides, and other oxidized species) formed. We employed model peptides to assess adduction reaction conditions and their effect on product formation, coupled with *in silico* quantum mechanical calculations to predict relative reactivity of electrophilic AA metabolites with selected protein nucleophiles. In addition, we examined protein adduction by both MS-based whole protein analysis and bottom-up proteomic assessment. Our findings confirm earlier work showing that sulfinamide and sulfonamide adducts are specifically formed at ^93^Cys of human β-Hb. In addition, we demonstrate that ^104^Cys of α-Hb and ^112^Cys of β-Hb can also undergo adduction under certain reaction conditions. CD and UV-Vis analysis revealed that the cysteine adductions induced structural changes in Hb, including Hb deoxygenation and the loss of α-helical content. The results provide additional understanding of the covalent interaction of aromatic amine metabolites with protein nucleophiles and the potential for physicochemical changes associated with thiol adduction in Hb.

## Results

### Quantum Mechanical Calculations

The calculated parameters associated with softness/hardness and relative electrophilicity/nucleophilicity of AA metabolites and potential target protein nucleophiles are shown in Tables 1 and 2. The data in Table 1 indicate that NOB is predicted to be a softer and more electrophilic species than PhNHOH, based on the relative values for σ and ω (0.346 eV^−1^ and 1.32 eV, respectively, for PhNHOH and 0.611 eV^−1^ and 5.71 eV, respectively, for NOB). For target nucleophiles, the data indicate that the Cys thiolate is expected to be a softer nucleophile than either the protonated (neutral) Cys thiol form or neutral forms of His and Lys. Table 2 presents data on the calculated reactivity index (ω^−^) for each electrophilic species with relevant protein nucleophiles. The reactivity index for Cys thiolate with either electrophile (1.03 eV and 3.18 eV for PhNHOH and NOB, respectively) was found to be higher than for the neutral thiol or for unprotonated His and Lys. In addition, NOB exhibited a higher ω^−^ when paired with Cys (−1) than did PhNHOH.

**Table 1:** Calculated quantum mechanical parameters for aniline derived electrophiles and biological nucleophiles.

**Electrophile**	***E***_**LUMO**_ **(eV)**	***E***_**HOMO**_ **(eV)**	**µ (eV)** ^**a**^	**η (eV)**	**σ (eV** ^**−1**^ **)**	**ω (eV)**
PhNHOH	0.13	−5.65	−2.76	2.89	0.346	1.32
NOB	−2.69	−5.96	−4.32	1.64	0.611	5.71
**Nucleophile**						
Cys (0)^b^	−0.16	−7.01	−3.59	3.43	0.292	
Cys (−1)	4.29	0.07	2.18	2.11	0.474	
His (0)	−0.39	−5.87	−3.13	2.74	0.365	
Lys (0)	0.10	−6.26	−3.08	3.18	0.314	

^a^Abbreviations: μ, chemical potential; η, chemical hardness; σ, chemical softness; ω, electrophilicity index (calculated for electrophiles only).

^b^Ionization state of nucleophilic atom.

**Table 2:** Calculated nucleophilicity (reactivity) index for reaction of biological nucleophiles with aniline derived electrophiles.

**Electrophile**	**Nucleophile**	**ω** ^**−**^ **(eV)** ^***a***^
PhNOH	Cys (0)^*b*^	0.03
	Cys (−1)	1.03
	His (0)	0.01
	Lys (0)	0.00
NOB	Cys (0)	0.04
	Cys (−1)	3.18
	His (0)	0.10
	Lys (0)	0.11

^a^ω^−^, reactivity index.

^b^Ionization state of nucleophilic atom.

### PhNHOH- and NOB-adducted peptides

*In vitro* incubation with various peptides containing nucleophilic residues was used to determine if only free thiols are modified by PhNHOH or NOB. To this end, the synthetic peptides PAAKAA, PAACAA, and PAAHAA, in addition to human acetyl ACTH (1–17), and acetyl γ-endorphin were subject to adduction. The latter two peptides contain no Cys residues but do include potentially reactive Lys and His residues. Synthetic peptides were acetylated at the N-terminus and amidated at the C-terminus. Even with high amine:peptide molar excess, the only adducted peptide obtained was PAACAA, with modification at the Cys residue confirmed by MS/MS analysis. MS/MS ion fragmentation data for the modified PAACAA peptide are summarized in Table 3 and MS/MS spectra are shown in Supplementary Information Figure S1-A. In contrast to Cys modification, no adduction at Lys or His was identified with either PhNHOH or NOB.

**Table 3:** MS/MS ion series of adducted peptides with PhNHOH and NOB.

**Peptide**	**Unadducted** ***m/z*** **[M+H]** ^**+**^	**Cys-Sulfinamide** ***m/z*** **[M+H]** ^**+**^	**Ions detected (** ***m/z*** **)** ^***a***^
	544.5	651.2	112.1 (a_1_), 140.1 (b_1_), 160.8 (y_2_), 211.0 (b_2_), 253.6 (a_3_), 281.9 (b_3_), **350.9 (y**_**4**_ **-[NHPh])**, **421.9 (y**_**5**_ **-[NHPh])**
	857.2	964.3	110.0 (H), 126.0 (K), 128.9 (K), 138.0 (H), 225.1 (b_2_+H_2_O), 243.2 (b_2_), 260.1 (y_2_), 357.2 (y_3_-H_2_O), 362.0 (b_3_-H_2_O), 375.0 (y_3_), 380.2 (b_3_), **562.2 (a**_**4**_ **)**, 610.2 **(b**_**5**_ **-[Ph]-H**_**2**_ **O)**, **628.0 (b**_**5**_ **-[Ph])**, **788.1 (a**_**6**_ **-NH**_**3**_ **)**, **869.0 ([M+H]** ^**+**^ **-[Ph]-H**_**2**_ **O)**, **870.2 ([M + H]** ^**+**^ **-[Ph]-NH**_**3**_ **)**

^a^The primary ions for both peptides were singly charged. [Ph] = loss of phenyl group during fragmentation; [NHPh] = loss of –NH-Ph group during fragmentation; H = histidine, K = lysine. Peptide fragmentation indicated using standard peptide ion nomenclature^76^. The calculated *m/z* were obtained using Protein Prospector^77^. “b” and “y” = fragments corresponding to the cleavage of the peptide bond; “a” = fragments corresponding to the cleavage of the bond between the Cα and C=O of each amino acid. Ions shown in bold are confirmatory of specific cysteine modification. Product ion spectra are shown in Supplementary Information Figure S1.

**Figure 2:**
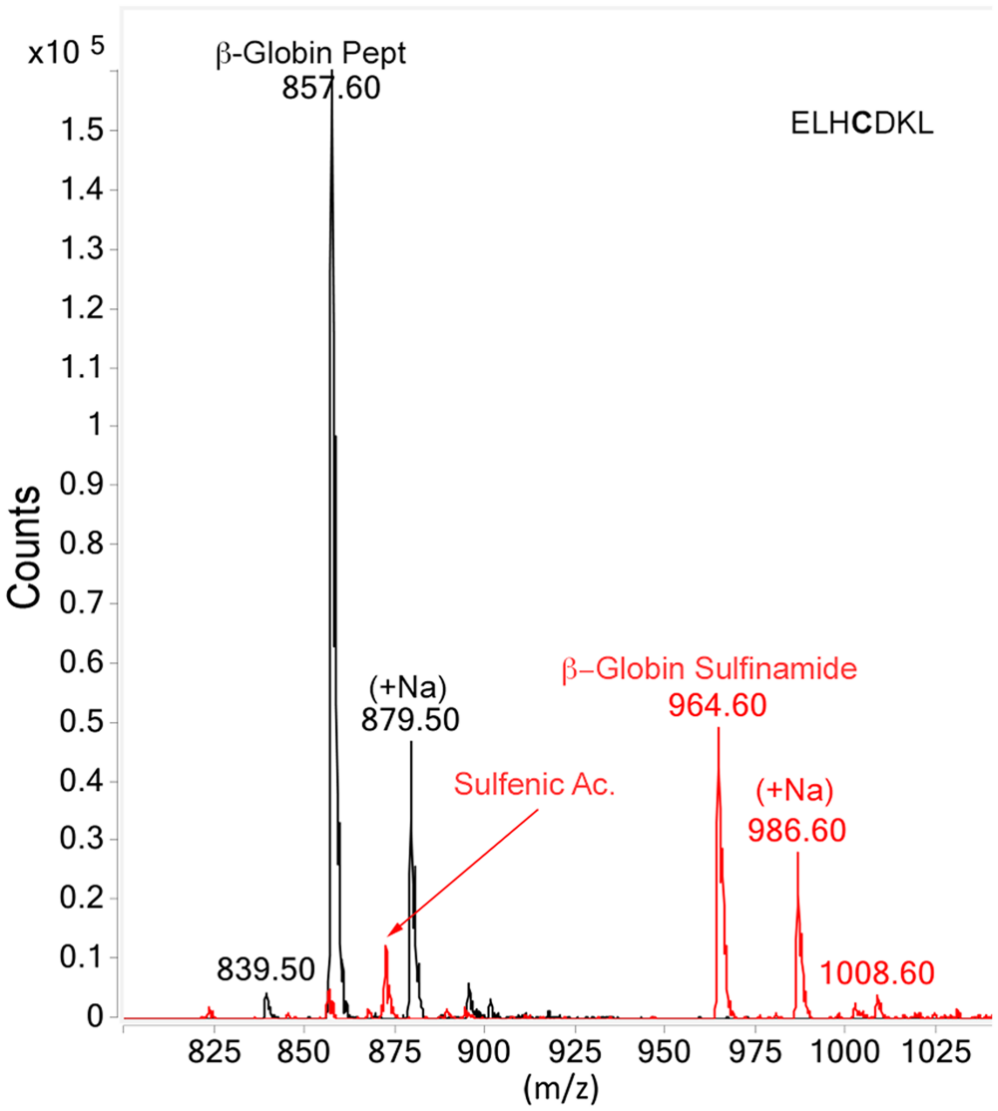
β-Globin peptide moiety mass spectra. Black trace corresponds to the native peptide and red trace corresponds to adducted peptide. Adducted β-globin peptide sulfinamide peak corresponds to a mass increase of 107 Da compared to control. Note stoichiometric conversion of Cys indicated by lack of peak for unmodified peptide in treated sample.

Additionally, adduction of a synthetic heptapeptide corresponding to residues 90–96 of human β-Hb (ELHCDKL), which contains free Cys, His, and Lys, was also examined (Table 3). Figure 2 illustrates MS data for the control and adducted peptides. The major product of adduction was the sulfinamide, as verified by a molecular mass increment of +107 Da, with minor amounts of sulfenic acid modification. Cys sulfinamide formation was also confirmed by MS/MS analysis (Table 1; Supplementary Information Figure S1-B). The pH dependence of adduction of the β-globin peptide was assessed in the range of 6.5 to 9.0 in 0.5 pH increments (data not shown). Adduction was first detected at pH 7.0; maximum adduction was noted at pH 8.5 and higher. Based on these data and to minimize protein structural changes that might occur in more alkaline solutions, pH 7.8 was chosen for all further experiments. Time course studies indicated that maximal peptide adduction was obtained with incubation times longer than 25 min (data not shown).

**Figure 3:**
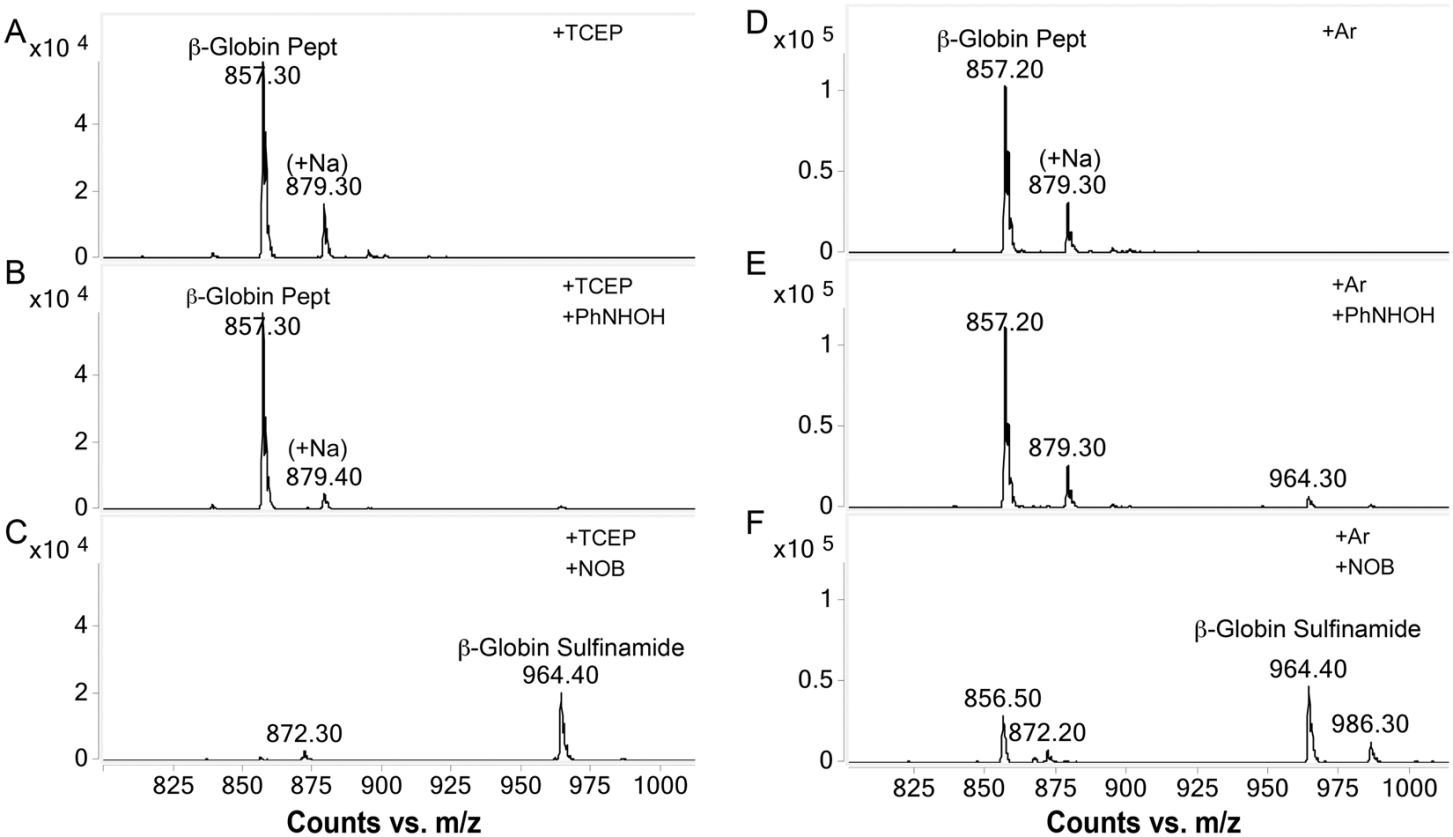
Adduction of the β-globin peptide under reducing conditions with TCEP (**A–C**) and under anaerobic (argon atmosphere) conditions (**D–F**). (**A**) Mass of 857 Da corresponds to the unmodified β-globin peptide, (**B**) No adduction was observed when the peptide was treated with TCEP followed by PhNHOH, (**C**) Peak for sulfinamide modified peptide (964 Da) was observed when peptide was treated with TCEP followed by NOB. Similar results were noted for samples without reducing agent but incubated under anaerobic conditions (**D–F**).

Experiments under reducing/alkylating and aerobic/anaerobic conditions were then performed to establish whether these environments could affect thiol adduction and to determine the stability of the sulfinamide formed. MS-based mass increment studies (Fig. 3) demonstrated that no adduction occurred on the β-globin peptide when incubation with PhNHOH was performed in the presence of the reducing agent tris(2-carboxyethyl)phosphine (TCEP), which inhibited oxidation of PhNHOH to NOB (Fig. 3B). Similarly, only very small amounts of sulfinamide were produced when incubation was performed without TCEP but under anaerobic conditions (Fig. 3E). In contrast, NOB was able to adduct peptide in either the presence of TCEP or under anaerobic conditions (Fig. 3C and F), confirming that the nitrosoarene can directly adduct thiols under reducing conditions.

### Modification of proteins by PhNHOH and NOB

**Figure 4:**
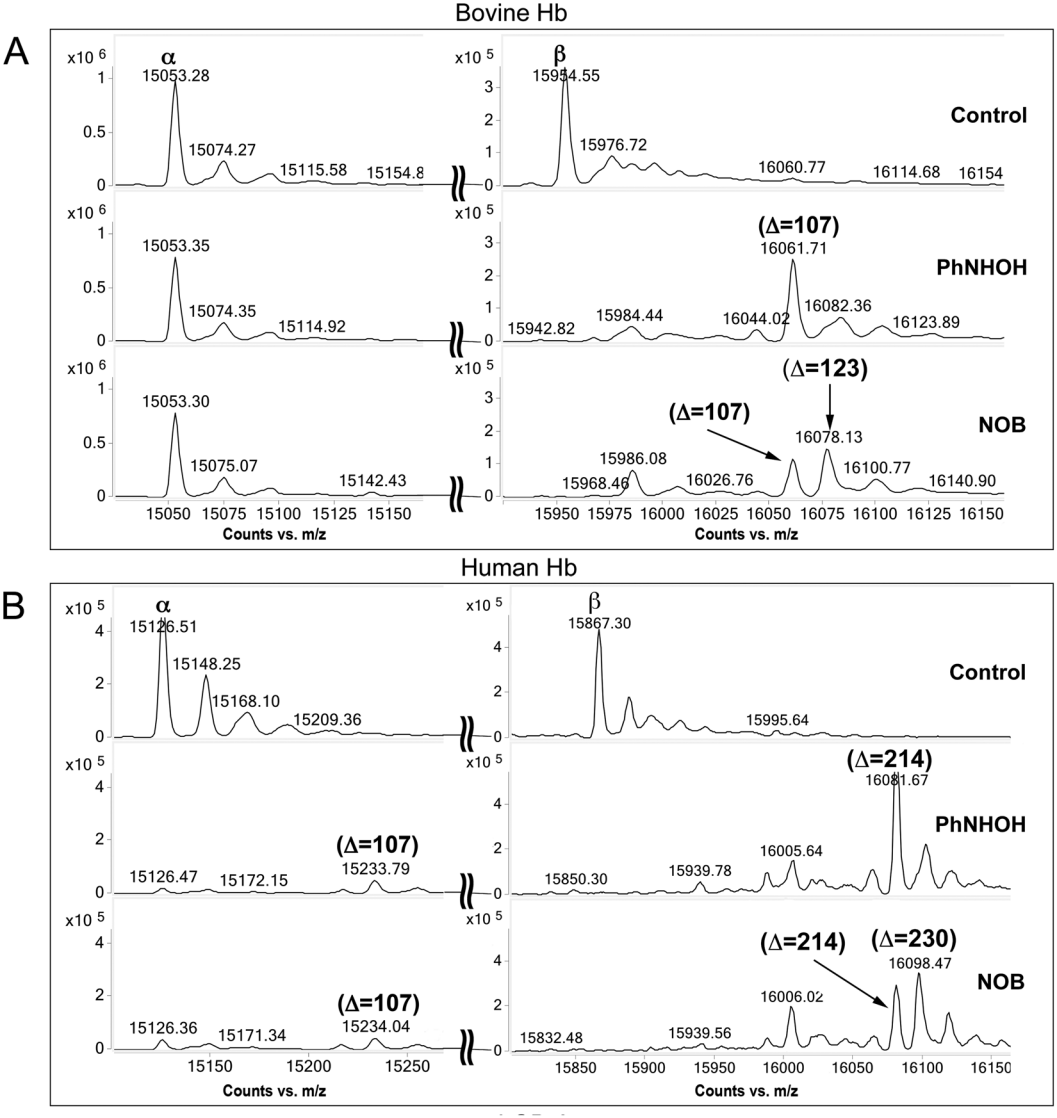
Adduction of human and bovine Hb proteins with PhNHOH and NOB. (**A**) No adduction of bovine Hb α-subunit (no free Cys residue) was observed with either PhNHOH or NOB (left panel). For bovine Hb β-subunit (free ^91^Cys) an increase of 107 Da corresponding to sulfinamide modification following PhNHOH is noted (right panel). Following NOB treatment, formation of one sulfinamide (+107 Da) and one sulfonamide (+123 Da) modification was observed. (**B**) For human Hb α-subunit (free ^104^Cys), treatment with either PhNHOH or NOB yields an increase of 107 Da corresponding to one sulfinamide modification (left panel). For human Hb β-subunit (free β^93^Cys and ^112^Cys), treatment with PhNHOH results in an increase of 214 Da, corresponding to two sulfinamide modifications (right panel). In the presence of NOB, a combination of one sulfinamide and one sulfonamide modification (+230 Da) was observed. Note stoichiometric conversion of bovine β-subunit and human α- and β-subunits with both compounds.

Initial studies indicated that protein adduction essentially reached completion after 5 h under the incubation conditions used. Based on these data, and with the goal of achieving stoichiometric modification of highly reactive free thiols in Hb, all protein experiments were conducted using overnight incubation with PhNOH or NOB. Analysis of whole protein by LC-QTOF-MS/MS (Fig. 4) following aromatic amine metabolite exposure provided the necessary mass resolution and accuracy to allow the direct detection of low molecular weight modifications in bovine and human Hb and β-LGB-A. Control proteins showed a measured mass accuracy within ±1–3 ppm. For bovine α-Hb and β-Hb, deconvoluted spectra showed molecular masses of 15053.28 Da and 15954.55 Da, respectively (Fig. 4A); whereas for human α-Hb and β-Hb, deconvoluted spectra showed molecular masses of 15126.51 Da and 15867.30 Da, respectively (Fig. 4B). Higher mass ion species noted in control protein preparations represent Na^+^ adducts (*e.g*., 15074.27 Da in control α-Hb).

A difference in mass (*i.e*., mass increment) between the adducted and control protein was consistent with the specific adduct formed and the stoichiometry of modification. Initial studies were conducted with bovine Hb, which has only one free Cys moiety (*i.e*., β^91^Cys; equivalent to human Hb β^93^Cys). After adduction with PhNHOH, a unit mass increment of +107 Da was observed, corresponding to a sulfinamide modification (theoretical mass increment +107.0371 Da). In contrast, adduction with NOB yielded two modifications on β^91^Cys; a sulfinamide (+107 Da) and a sulfonamide (+123 Da; theoretical mass increment +123.0320 Da) (Fig. 4A, right side). As expected, the lack of free thiol moieties in the bovine Hb α-subunit yielded unchanged molecular masses upon treatment (Fig. 4A, left side).

For the human Hb β-subunit, a 214 Da mass increase, consistent with two sulfinamide modifications, was observed with either chemical. Furthermore, upon reaction with NOB, masses consistent with the addition of one sulfinamide and one sulfonamide (+230 Da) were also observed (Fig. 4B, right side). Notably, human Hb β-subunit has two free thiols,^93^Cys and ^112^Cys; MS/MS results (discussed below) demonstrate that both cysteines were modified by these chemicals. Additionally, for the human Hb α-subunit, an increment of +107 Da, likely corresponding to sulfinamide formation on ^104^Cys, was observed with either chemical (Fig. 4B, left side). For both bovine and human Hb subunits, the absence of MS peaks corresponding to unmodified protein in treated preparations is consistent with stoichiometric modification of the free thiols. When the adducted bovine and human Hb were reincubated following removal of unreacted aromatic amine metabolite, it was observed that the sulfinamide and sulfonamide adducts were stable for at least one week (data not shown).

**Figure 5:**
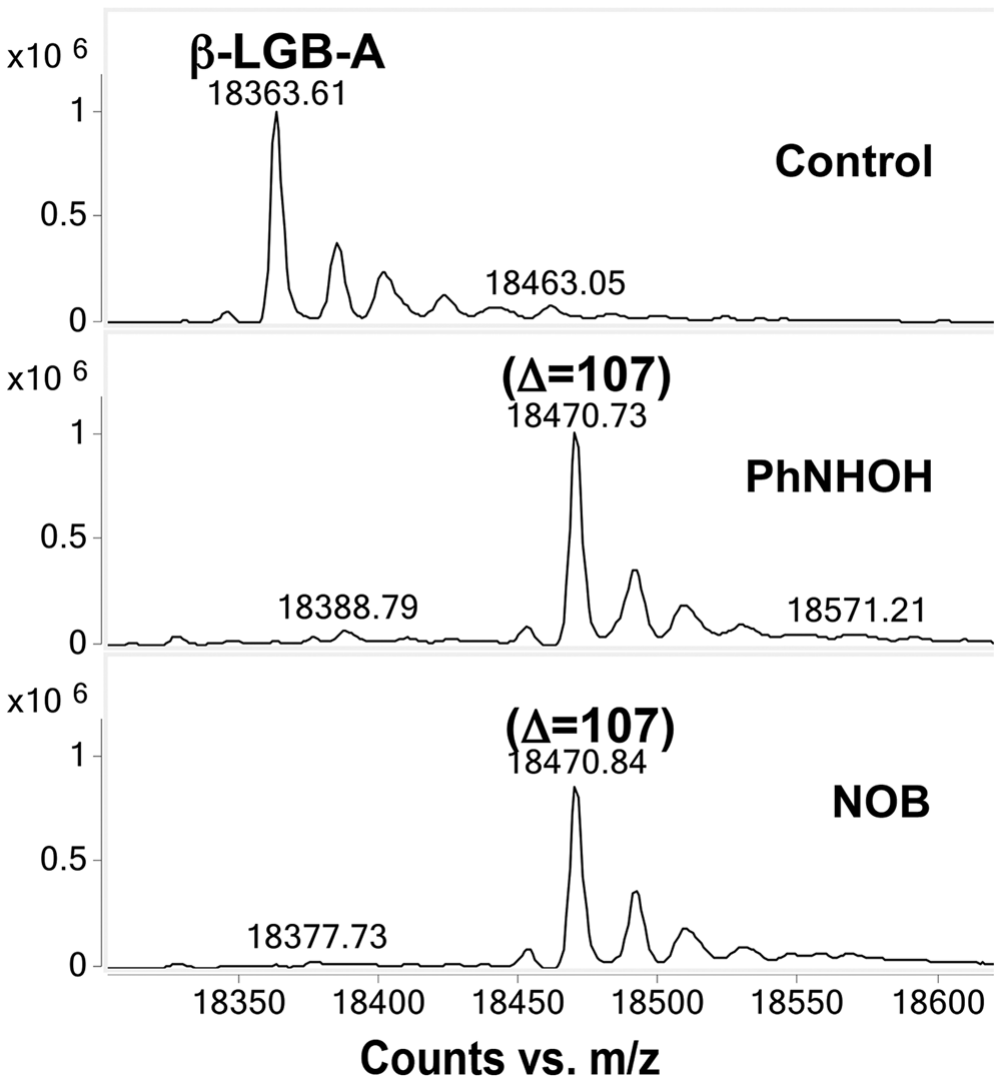
Bovine β-LGB-A adduction. After adduction with either PhNHOH or NOB, a mass increase of 107 Da was observed, which corresponds to sulfinamide formation on ^121^Cys. No sulfonamide derivative was observed. Note stoichiometric conversion of Cys indicated by lack of peak for unmodified protein in treated samples.

To determine if the presence of the heme group influenced the formation of the observed sulfonamide product, β-LGB-A, a protein without heme but containing a single free thiol (^121^Cys), was subjected to adduction. After treatment with PhNHOH or NOB, a +107 Da mass increment, corresponding to sulfinamide formation, was observed (Fig. 5). However, unlike in the case of the human Hb β-subunit, no sulfonamide modifications were observed for this protein in the presence of NOB. Stoichiometric modification was also observed for this protein. These results suggest that the presence of the heme group may facilitate oxidation of the sulfinamide to sulfonamide following direct adduction by the nitrosoarene species.

The accessibility to small electrophiles, such as aromatic amine metabolites, of thiols in human Hb was assessed by measuring reactivity towards the alkylating agent IAM. Only β^93^Cys was found to be susceptible to alkylation (mass increment +57 Da) (Supplementary Information Figures S2–B and S2–G). When human Hb was initially alkylated and then reacted with PhNHOH or NOB, sulfinamide formation was observed on the α-Hb subunit, along with one alkylated cysteine and one sulfinamide adduct in the β-Hb subunit (Supplementary Information Figures S2–D and S2–I). These data suggest that, while α^104^Cys and β^112^Cys, which are generally shielded in the folded protein^29,30^, were not modified by IAM, both PhNHOH and NOB are capable of modifying these thiols under the conditions used in this study.

### Identification of protein adduction sites

Adducted proteins were subjected to combinatorial Lys-C/trypsin digestion and high resolution MS/MS proteomic analysis to confirm the sites and types of modifications noted in the whole protein studies. The sequence coverage achieved for both protein subunits was 95 to 97% for the bovine Hb and 100% for human Hb. Table 4 and Supplemental Information Figures S3–S6 present complete MS/MS ion data and spectral interpretation for specific modified digested peptides detected in bovine and human Hb pre-treated with aromatic amine metabolites. Adducted peptide IDs were confirmed with very low mass error compared to calculated and with multiple b- and y-series ions for each peptide.

**Table 4:** Adduct ion series of the LysC/tryptic peptides from Hb adducted with PhNHOH or NOB. Calculated *m/z* values are in parentheses. Product ion spectra are shown in Supplementary Information Figures S3–S6.

Ions detected (m/z)
**Bovine** β**-Hb Sulfinamide (** ^**91**^ **Cys)** → **GTFAALSELHcDKLHVDPENFK [M**+**3H]** ^**3+**^ ** = 860.0861**130.08653 (130.08626) **[y**_**1**_ **]-NH**_**3**_ **;** 147.11298 (147.11281) **[y**_**1**_ **];** 288.13455 (262.13428) **[b**_**2**_ **]-H**_**2**_ **O;** 294.18146 (294.18123) **[y**_**2**_ **];** 377.18265 (377.18197) **[b**_**4**_ **];** 408.22513 (408.22416) **[y**_**3**_ **];** 430.209329 (430.20852) **[b**_**5**_ **]-H**_**2**_ **O;** 488.21426 (488.21430) **[b**_**13**_ **]** ^**3+**^ **-H**_**2**_ **O;** 505.24142 (505.24120) **[b**_**10**_ **]** ^**2+**^ **-H**_**2**_ **O;** 634.32001 (634.31953) **[y**_**5**_ **];** 749.34845 (749.34648) **[y**_**6**_ **];** 848.41724 (848.41490) **[M**+**3H]** ^**3+**^ **-NH**_**3**_ **-H**_**2**_ **O, [y**_**7**_ **];** 985.47913 (985.47381) **[y**_**8**_ **]**
**Bovine** β**-Hb Sulfinamide (** ^**91**^ **Cys)** → **HLDDLKGTFAALSELHcDK [M**+**4H]** ^**4+**^ ** = 556.60261**147.11319 (147.11289) **[y**_**1**_ **];** 155.08179 (155.08180) **[b**_**4**_ **]** ^**3+**^ **-H**_**2**_ **O;** 251.15051 (251.15038) **[b**_**2**_ **];** 262.14038 (262.13978) **[y**_**2**_ **];** 288.13510 (288.13521) **[b**_**8**_ **]** ^**3+**^ **-H**_**2**_ **O;** 366.7686 (366.77231) **[b**_**3**_ **];** 431.7241(431.7245) **[b**_**8**_ **]** ^**2+**^ **-H**_**2**_ **O;** 481.20435 (481.2041) **[b**_**4**_ **];** 500.26773 (500.2561) **[b**_**14**_ **]** ^**3+**^ **;** 514.26343 (514.2640) **[b**_**9**_ **]** ^**2+**^ **;** 540.7770 (540.7773) **[b**_**10**_ **]** ^**2+**^ **-H**_**2**_ **O;** 529.7815(549.7826) **[b**_**10**_ **]** ^**2+**^ **;** 576.29517 (576.2958) **[b**_**5**_ **]-H**_**2**_ **O, [b**_**11**_ **]** ^**2+**^ **-H**_**2**_ **O;** 585.30141 (585.30119) **[b**_**11**_ **]** ^**2+**^ **;** 594.28912 (594.28892) **[b**_**5**_ **];** 641.84326 (641.8431) **[b**_**12**_ **]** ^**2+**^; 722.38269 (722.3832) **[b**_**6**_ **]**; 740.87494 (740.8752) **[b**_**14**_ **]** ^**2+**^ **-H**_**2**_ **O;** 861.45563 (861.4576) **[y**_**15**_ **]** ^**2+**^ **-H**_**2**_ **O;** 880.45129 (880.4523) **[b**_**8**_ **]**
**Bovine** β**-Hb Sulfonamide (Cys** ^**91**^ **)** → **GTFAALSELHcDK [M**+**2H]** ^**2+**^ ** = 757.8521**130.08626 (130.08626) **[y**_**1**_ **]-NH**_**3**_ **;** 147.11314 (147.11281) **[y**_**1**_ **];** 262.13895 (262.13976) **[y**_**2**_ **];** 288.13467 (288.13428) **[b**_**2**_ **]-H**_**2**_ **O;** 306.14542 (306.14485) **[b**_**2**_ **];** 361.15082 (361.15042) **[y**_**5**_ **]** ^**2+**^ **-NH**_**3**_ **;** 377.18219 (377.18197) **[b**_**4**_ **];** 430.20862 (430.20852) **[b**_**5**_ **]-H**_**2**_ **O;** 488.18082 (488.18195) **[y**_**3**_ **];** 607.23022 (607.23030) **[y**_**4**_ **]-H**_**2**_ **O;** 625.24023 (625.240860) **[y**_**4**_ **];** 738.32526 (738.32493) **[y**_**5**_ **];** 867.36890 (867.36753) **[y**_**6**_ **];** 954.39948 (954.39956) **[y**_**7**_ **];** 1067.48242 (1067.48363) **[y**_**8**_ **];** 1138.52222 (1138.52075) **[y**_**9**_ **];** 1209.55701 (1209.55787) **[y**_**10**_ **]**
**Human** α**-Hb Sulfinamide (** ^**104**^ **Cys)** → **LLSHcLLVTLAAHLPAEFTPAVHASLDK [M**+**4H]** ^**4+**^ ** = 769.6699**120.08102 (120.08100) **[y**_**3**_ **]** ^**3+**^ **-NH**_**3**_ **;** 147.11282 (147.11280) **[y**_**1**_ **];** 227.17560 (227.17542) **[b**_**2**_ **];** 251.15024 (251.15020) **[y**_**7**_ **]** ^**3+**^ **-H**_**2**_ **O;** 262.13995 (262.13970) **[y**_**2**_ **];** 335.67963 (335.67960) **[y**_**6**_ **]** ^**2+**^ **;** 451.26715 (451.26636) **[b**_**4**_ **];** 462.25528 (462.25586) **[y**_**4**_ **];** 469.25836 (469.25840) **[y**_**9**_ **]** ^**2+**^ **;** 519.78263 (519.78260) **[y**_**10**_ **]** ^**2+**^ **;** 533.29260 (533.29298) **[y**_**5**_ **];** 670.35083 (670.35080) **[y**_**6**_ **]**, 671.35455 (671.35189) **[b**_**23**_ **]** ^**4+**^ **-H**_**2**_ **O;** 741.87897 (741.87900) **[y**_**14**_ **]** ^**2+**^ **;** 769.42078 (769.42031) **[b**_**21**_ **]** ^**3+**^ **, [M** + **4 H]** ^**4+**^ **, [y**_**7**_ **];** 840.45758 (840.45743) **[y**_**8**_ **];** 919.49902 (919.49900) **[y**_**9**_ **]-H**_**2**_ **O;** 937.51050 (937.5102) **[y**_**9**_ **];** 938.51270 (938.51202) **[b**_**26**_ **]** ^**3+**^ **;** 994.53198 (994.53000) **[y**_**19**_ **]** ^**2+**^ **;** 1038.55872 (1038.55788) **[y**_**10**_ **];** 1185.62183 (1185.62630) **[y**_**11**_ **];** 1314.66992 (1314.66890) **[y**_**12**_ **];** 1385.71204 (1385.70602) **[y**_**13**_ **];** 1464.74792 (1464.73224) **[y**_**14**_ **]-H**_**2**_ **O;** 1482.75623 (1482.75879) **[y**_**14**_ **]**
**Human** α**-Hb Sulfonamide (** ^**104**^ **Cys)** → **LLSHcLLVTLAAHLPAEFTPAVHASLDK [M**+**4H]** ^**4+**^ ** = 773.9194**120.08108 (120.08100) [**y**_**3**_ **]** ^**3+**^ **-NH**_**3**_ **;** 130.08655 (130.10250) **[y**_**1**_ **]-NH**_**3**_ **;** 147.11325 (147.11280) **[y**_**1**_ **];** 227.17562 (227.17542) **[b**_**2**_ **];** 251.15022 (251.15020) **[y**_**7**_ **]** ^**3+**^ **-H**_**2**_ **O;** 262.14050 (262.13970) **[y**_**2**_ **];** 365.67911 (365.67914) **[y**_**6**_ **]** ^**2+**^ **;** 462.25568 (462.25586) **[y**_**4**_ **];** 469.25897 (469.25850) **[y**_**9**_ **]** ^**2+**^ **;** 477.26245 (477.26282) **[b**_**17**_ **]** ^**4+**^ **;** 533.29346 (533.29298) **[y**_**5**_ **];** 652.32514 (652.32401) **[y**_**6**_ **]-H**_**2**_ **O;** 670.35242 (670.35189) **[y**_**6**_ **];** 671.35455 (671.35189) **[b**_**23**_ **]** ^**4+**^ **-H**_**2**_ **O;** 677.30865 (677.30855) **[b**_**5**_ **];** 748.40765 (748.40811) **[b**_**13**_ **]** ^**2+**^ **;** 769.420141 (769.42031) **[y**_**7**_ **];** 805.45166 (805.45290) **[y**_**23**_ **]** ^**3+**^ **;** 840.45911 (840.45743) **[y**_**8**_ **];** 903.47278 (903.47669) **[b**_**7**_ **];** 937.51050 (937.51020) **[y**_**9**_ **];** 953.51794 (953.51642) **[b**_**17**_ **]** ^**2+**^ **;** 1002.54315 (1002.54511) **[b**_**8**_ **];** 1038.55408 (1038.55788) **[y**_**10**_ **];** 1085.57825 (1085.58222) **[b**_**9**_ **]-H**_**2**_ **O, [y**_**21**_ **]** ^**2+**^ **-H**_**2**_ **O;** 1185.62427 (1185.62630) **[y**_**11**_ **];** 1287.71313 (1287.71398) **[b**_**11**_ **];** 1314.67395 (1314.66890) **[y**_**12**_ **];** 1358.74841 (1358.75110) **[b**_**12**_ **];** 1465.75183 (1465.75324) **[y**_**14**_ **]-NH**_**3**_ **;** 1482.75415 (1482.75879) **[y**_**14**_ **]**
**Human** β**-Hb Sulfinamide (Cys** ^**93**^ **)** → **GTFATLSELHcDKLHVDPENFR [M**+**4H]** ^**4+**^ ** = 660.0722**159.07665 (159.07643) **[b**_**2**_ **];** 175.11937 (175.11896) **[y**_**1**_ **];** 287.13516 (287.13432) **[y**_**7**_ **]** ^**3+**^ **-NH**_**3**_ **;** 288.13461 (287.13428) **[b**_**3**_ **]-H**_**2**_ **O;** 436.23087 (436.23031) **[y**_**3**_ **];** 507.24530 (507.24531) **[y**_**8**_ **]** ^**2+**^ **;** 507.74261 (507.73940) **[y**_**16**_ **]** ^**4+**^ **-H**_**2**_ **O;** 660.35339 (660.35340) **[b**_**7**_ **]-H**_**2**_ **O;** 662.32538 (662.32568) **[y**_**5**_ **];** 685.34485 (685.34661) **[b**_**18**_ **]** ^**3+**^ **-H**_**2**_ **O, [y**_**11**_ **]** ^**2+**^ **;** 720.36182 (720.35495) **[y**_**17**_ **]** ^**3+**^ **;** 777.34784 (777.34780) **[y**_**6**_ **];** 876.42334 (876.42105) **[y**_**7**_ **];** 1013.48010 (1013.47996) **[y**_**8**_ **];** 1014.48511 (1014.47390) **[y**_**16**_ **]** ^**2+**^ **-H**_**2**_ **O**
**Human** β**-Hb Sulfonamide (Cys** ^**93**^ **)** → **GTFATLSELHcDK [M**+**2H]** ^**2+**^ ** = 772.8585**142.11299 (142.09884) **[y**_**1**_ **];** 159.07658 (159.07643) **[b**_**2**_ **];** 262.13950 (262.13976) **[y**_**2**_ **];** 288.13455 (288.13428) **[b**_**3**_ **]-H**_**2**_ **O;** 306.14536 (306.14485) **[b**_**3**_ **];** 460.22028 (460.21908) **[b**_**5**_ **]-H**_**2**_ **O;** 488.18103 (488.18195) **[y**_**3**_ **];** 607.22906 (607.23030) **[y**_**4**_ **]-H**_**2**_ **O;** 625.24030 (625.24086) **[y**_**4**_ **];** 738.32324 (738.32493) **[y**_**5**_ **];** 867.36823 (867.36753) **[y**_**6**_ **];** 954.39874 (954.39956) **[y**_**7**_ **];** 1067.48303 (1067.48363) **[y**_**8**_ **];** 1168.53088 (1168.53131) **[y**_**9**_ **];** 1239.56543 (1239.56843) **[y**_**10**_ **]**
**Human** β**-Hb Sulfinamide (Cys** ^**112**^ **)** → **LLGNVLVcVLAHHFGK [M**+**4H]** ^**4+**^ ** = 457.5087**147.11280 (147.11281) **[y**_**1**_ **];** 204.13460 (204.13428) **[y**_**2**_ **], [b**_**6**_ **]** ^**3+**^ **;** 209.10358 (209.11185) **[y**_**5**_ **]** ^**3+**^ **;** 227.17575 (227.17542) **[b**_**2**_ **];** 284.19692 (284.19689) **[b**_**3**_ **];** 313.16393 (313.16401) **[y**_**5**_ **]** ^**2+**^ **;** 348.68251 (348.68246) **[y**_**6**_ **]** ^**2+**^ **;** 351.20282 (351.20270) **[y**_**3**_ **];** 398.24048 (398.23982) **[b**_**4**_ **];** 405.22456 (405.22543) **[y**_**7**_ **]** ^**2+**^ **;** 454.7581 (454.7587) **[y**_**8**_ **]** ^**2+**^ **;** 480.28214 (480.28169) **[b**_**5**_ **]-NH**_**3**_ **;** 488.26184 (488.26161) **[y**_**4**_ **];** 497.30853 (497.30824) **[b**_**5**_ **];** 593.36572 (596.36576) **[b**_**6**_ **]-NH**_**3**_ **;** 609.3160 (609.29397) **[y**_**10**_ **]** ^**2+**^ **;** 610.39319 (610.39231) **[b**_**6**_ **];** 625.32111 (625.32052) **[y**_**5**_ **];** 696.35803 (696.35764) **[y**_**6**_ **];** 809.44238 (809.44171) **[y**_**7**_ **];** 908.51117 (908.51013) **[y**_**8**_ **]**
**Human** β**-Hb Sulfonamide (Cys** ^**112**^ **)** → **LLGNVLVcVLAHHFGK [M**+**3H]** ^**3+**^ ** = 615.3419**130.08650 (130.08626) **[y**_**1**_ **]-NH**_**3**_ **;** 147.11283 (147.11281) **[y**_**1**_ **];** 204.13448 (204.13428) **[y**_**2**_ **], [b**_**6**_ **]** ^**3+**^ **;** 209.11179 (209.11185) **[y**_**5**_ **]** ^**3+**^ **;** 227.17557 (227.17542) **[b**_**2**_ **];** 284.19699 (284.19689) **[b**_**3**_ **];** 351.20251 (351.20270) **[y**_**3**_ **];** 488.26111 (488.26161) **[y**_**4**_ **];** 567.78288 (567.7793) **[y**_**9**_ **]** ^**2+**^ **;** 615.34193 (615.30864) **[M** + **3 H]** ^**3+**^ **;** 617.31439 (617.31357) **[y**_**10**_ **]** ^**2+**^ **;** 625.31995 (625.32052) **[y**_**5**_ **];** 673.8555 (673.8551) **[y**_**11**_ **]** ^**2+**^ **;** 696.35712 (696.35764) **[y**_**6**_ **];** 800.40979 (800.42328) **[y**_**14**_ **]** ^**2+**^ **-NH**_**3**_ **;** 808.92328 (808.92197) **[y**_**14**_ **]** ^**2+**^ **;** 809.43182 (809.44171) **[y**_**7**_ **];** 865.45984 (865.46400) **[y**_**15**_ **]** ^**2+**^ **;** 908.50574 (908.51013) **[y**_**8**_ **];** 1134.55054 (1134.55132) **[y**_**9**_ **];** 1233.61816 (1233.62074) **[y**_**10**_ **];** 1346.69788 (1346.70481) **[y**_**11**_ **]**

For bovine Hb adducted by PhNHOH, the MS/MS fragmentation pattern confirmed the presence of a sulfinamide adduct on β^91^Cys (Table 2 and Figure S3-A). Following adduction with NOB, both sulfinamide and sulfonamide adducts were identified at β^91^Cys, (Table 4 and Figure S3-B,C). Among the digested peptides of human Hb, both sulfinamide and sulfonamide modifications were also found on α^104^Cys (Table 4 and Figure S4). Additional sulfinamide and sulfonamide modifications were confirmed at β^93^Cys and β^112^Cys of human Hb, as evident from MS/MS analysis of the corresponded digested peptide (Table 4 and Figures S5 and S6).

### Exposure of whole blood to PhNHOH and NOB

**Figure 6:**
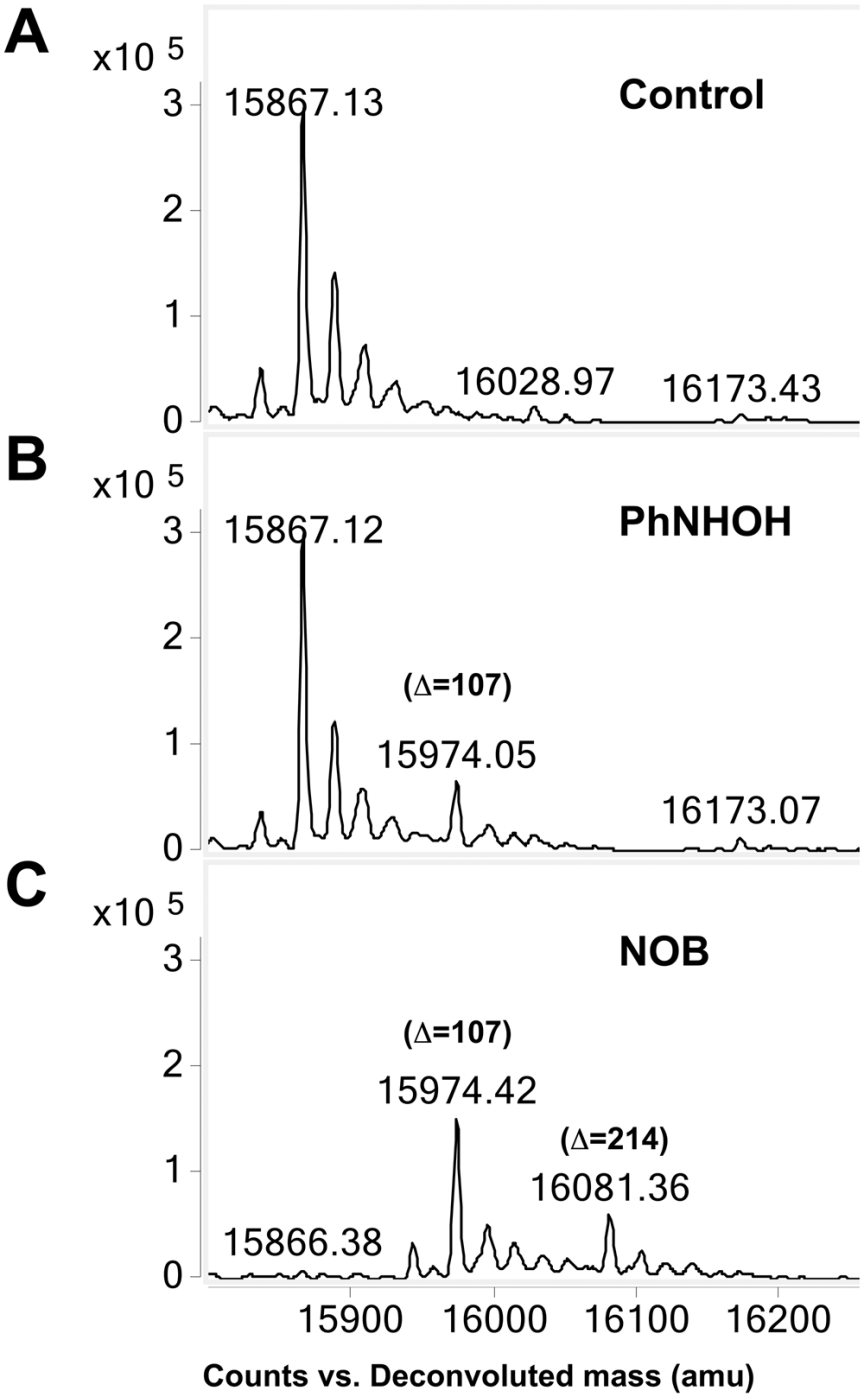
Whole blood exposure to PhNHOH and NOB. Human whole blood samples were exposed to PhNHOH and NOB and Hb subsequently purified and analyzed by ESI-QTOF-MS. Deconvoluted masses for the β-subunit are shown: (**A**) Human Hb extracted from untreated whole blood. (**B**) Whole blood exposed to PhNHOH; sulfinamide adducted β-Hb (15974 Da; +107 Da) is observed as a reaction product. (**C**) Whole blood exposed to NOB; modifications consistent with one (+107 Da) and two (+214 Da) sulfinamides were observed on the β-subunit.

To assess whether other blood components could influence the adduction process, heparinized human blood samples were treated with PhNHOH and NOB. Hb was then purified and mass increments due to adduction were assessed by whole protein MS as described earlier. In this experiment, β-Hb modified with one sulfinamide product (+107 Da) was observed after exposure to PhNHOH (Fig. 6B). In contrast, when blood was exposed to NOB, modified β-Hb with mass increments of both +107 and +214 Da, consistent with formation of one and two sulfinamides, respectively, was observed (Fig. 6C). In addition, we observed a peak at 15,233 Da for α-Hb, consistent with a single sulfinamide modification on ^104^Cys of this subunit (data not shown). In contrast to studies with purified Hb, sulfonamide products were not detected following whole blood incubations. Finally, stoichiometric conversion of free thiols in human β-Hb was noted with NOB, but not PhNHOH treatment (Fig. 6B and C). Identical results were observed when isolated RBCs (as opposed to whole blood) were exposed to either chemical.

### Human Hb adduction in the presence of GSH/GSSG

**Figure 7:**
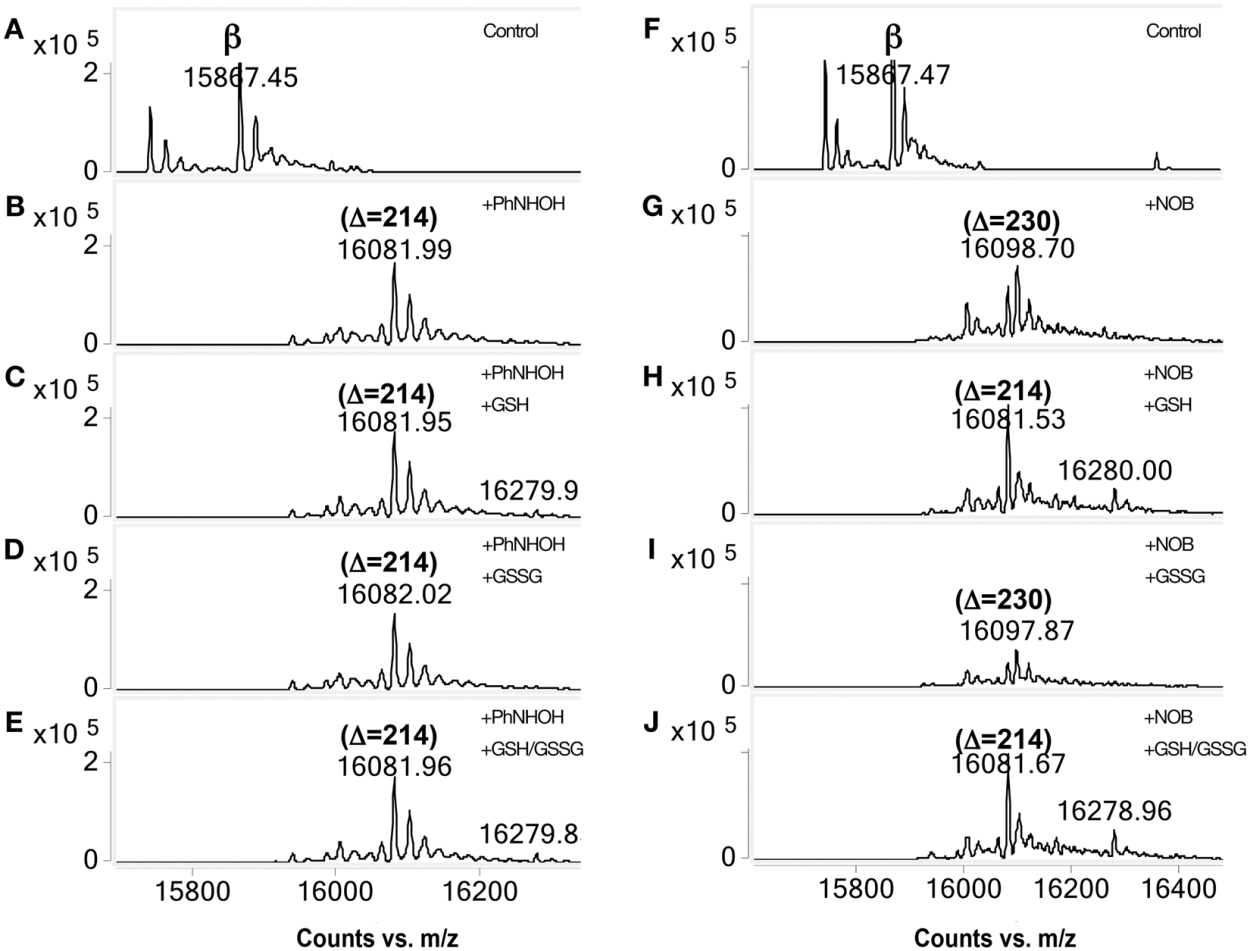
Adduction of human Hb β-subunit in the presence of GSH/GSSG. Left panel corresponds to the adduction with PhNHOH and the right panel to the adduction with NOB. (**A**) Human Hb control, (**B**) Human Hb incubated with PhNHOH, (**C**) Human Hb incubated with PhNHOH in presence of GSH, (**D**) Human Hb incubated with PhNHOH in presence of GSSG, (**E**) Human Hb incubated with PhNHOH in presence of both GSH and GSSG. Presence of GSH and/or GSSG does not affect sulfinamide (+214) formation. Right panel (**F–H**), represents the same experiment but with NOB. In the presence of GSH, no sulfonamide is formed (Compare **H** and **J** with **G** and **I**).

The lack of formation of sulfonamides observed in Hb following treatment of whole blood may have been due to the presence of other blood components that prevented sulfinamide oxidation, such as small thiols including GSH. Consequently, we carried out the adduction of purified human Hb in the presence of 1 mM reduced GSH, to model typical intracellular concentrations which reportedly range from 0.5 to 10 mM^31^. As in the whole blood experiments, only Cys sulfinamide modifications were observed for Hb treated with either PhNHOH or NOB in the presence of either GSH or a mixture of GSH and GSSG (Fig. 7). In contrast, the addition of oxidized glutathione GSSG alone did not have any effect on the adduction process with either PhNHOH or NOB. Control Hb in the presence of GSH, GSSG and GSH/GSSG did not exhibit changes in molecular mass (data not shown). These results suggest that the presence of small thiols in whole blood can inhibit the formation of products other than the sulfinamide.

### *In vitro* dose response for adduct formation in human Hb

**Figure 8:**
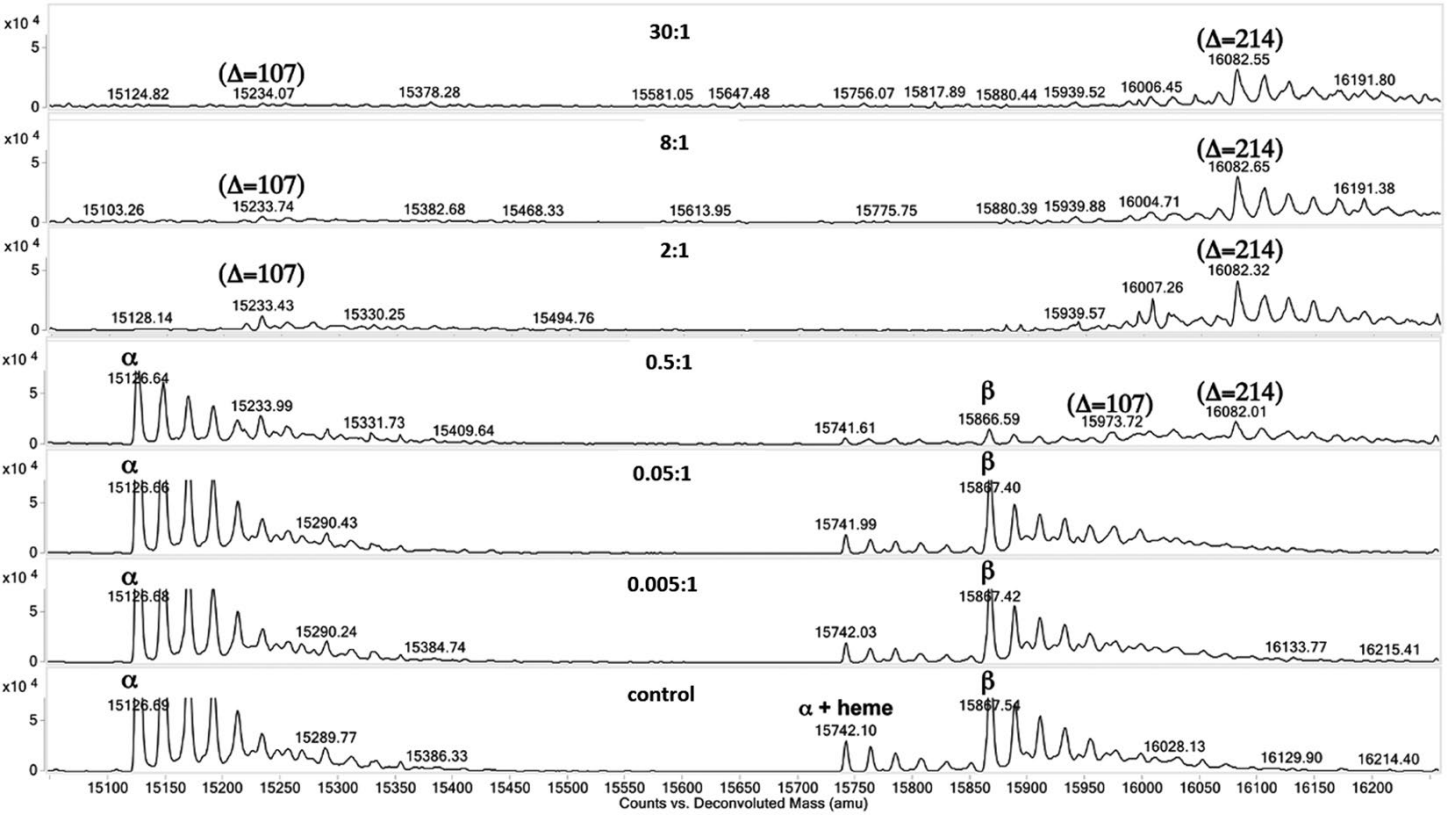
LC/ESI-MS spectra of adduction of human Hb with different molar ratios of PhNHOH. Human Hb concentration was 0.036 mM. PhNHOH:protein molar ratios were: 30:1 (1.08 mM), 8:1 (0.29 mM), 2:1 (0.07 mM), 0.5:1 (0.018 mM), 0.05:1 (0.002 mM), and 0.005:1 (0.0002 mM). For Hb α-subunit (free ^104^Cys), treatment with PhNHOH at molar ratios between 2:1 and 30:1 yielded a mass increase of 107 Da corresponding to one sulfinamide modification. For Hb β-subunit (free ^93^Cys and ^112^Cys), treatment with PhNHOH at molar ratios between 2:1 and 30:1 yielded in an increase of 214 Da corresponding to two sulfinamide modifications. Treatment with 0.5:1 ratio yielded a combination of the unadducted protein subunits, the adduction of the α-subunit with an increase of 107 Da and the adduction of both free cysteines of β-subunit with an increase of 214 Da. No adductions of the Hb subunits were observed at lower molar of 0.05:1 and 0.005:1.

It was also of interest to assess the potential of lower concentrations of AA metabolites to induce sulfinamide adduct formation in human Hb. Accordingly, incubation of Hb at metabolite:protein molar ratios ranging from 0.005:1 to 30:1, followed by MS-based whole protein mass increment analysis, was performed. For PhNHOH, molar ratios of 2:1 and higher yielded a mass increment of +107 Da for α-Hb, consistent with formation of a sulfinamide adduct at α^104^Cys, and a mass increment of +214 Da for β-Hb, consistent with formation of sulfinamide adducts at β^93^Cys and β^112^Cys as previously observed (Fig. 8). At a molar ratio of 0.5:1, PhNHOH treatment yielded a mix of unmodified and adducted products, while at the lowest molar ratios (0.05:1 and 0.005:1), no adduction was observed. Similar results were obtained with NOB, except that adduct levels were noted to be higher at the 0.5:1 molar ratio as compared to PhNHOH (data not shown).

### Circular Dichroism and UV/Vis spectroscopy

**Figure 9:**
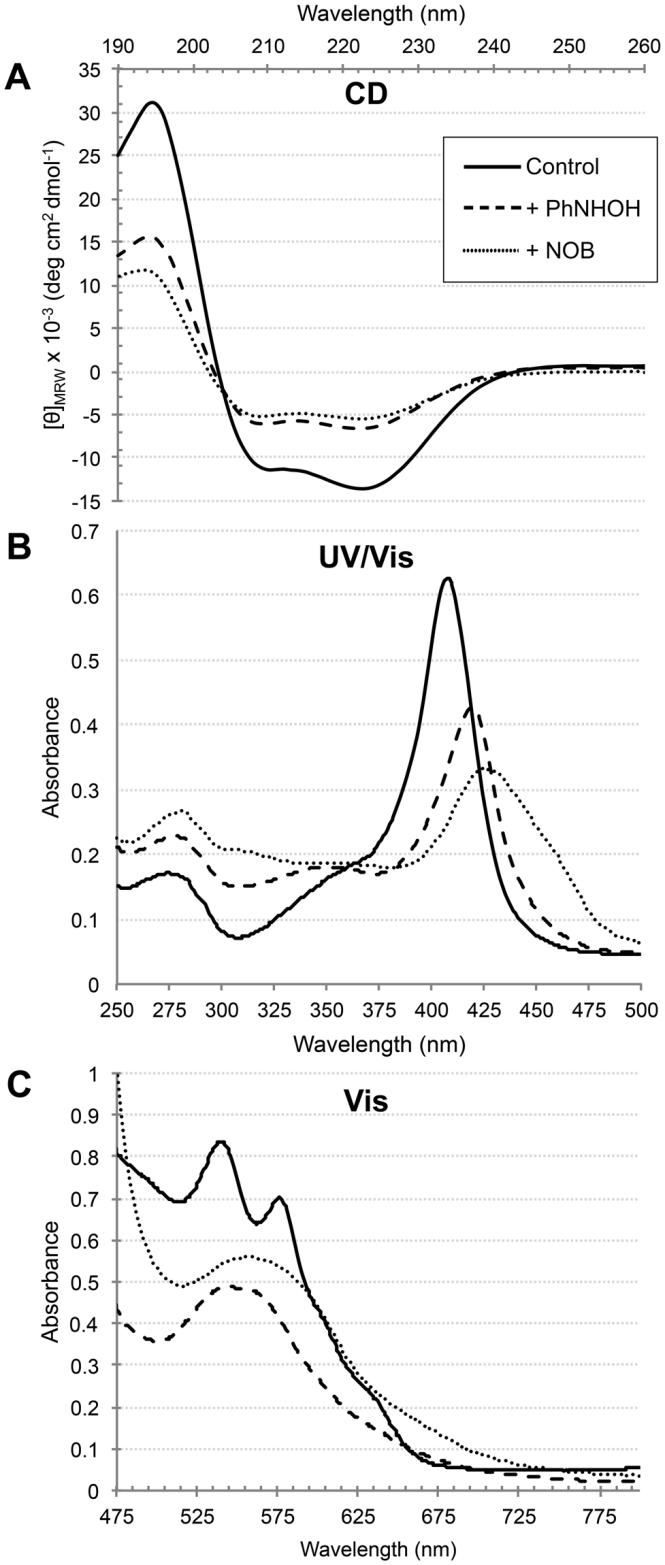
CD and UV/Vis spectroscopy of human Hb following treatment with PhNHOH and NOB. (**A**) CD spectra. A decrease in helicity is observed with both chemical compounds. Secondary structure α-helix contents are 47.7% for the control, 24.4% in presence of PhNHOH, and 21.1% in presence of NOB (see Supplementary Information Table S1). (**B**) UV/Vis absorbance spectra from 250–500 nm (Soret region). Adduction of Hb results in a hypochromic red shift in the maximal absorbance. (**C**) Visible absorbance spectra. Both compounds caused the disappearance of the double peaks of absorption at λ = 541 nm and λ = 578 nm, which are characteristic of oxygenated Hb. These are replaced by a single maximum; λ = 543 nm and λ = 556 nm for Hb adducted by PhNHOH and NOB respectively. The obtained results indicate that the Hb loses bound oxygen during the adduction process.

CD spectral analysis in the 190–260 nm range was carried out to detect conformational changes in human Hb induced by PhNHOH and NOB adduction. Control human Hb revealed a characteristic α-helix secondary structure with a π → π* positive perpendicular transition with a maximum at λ = 192 nm and two negative minima at λ = 208 and 222 nm, which correspond to the π → π* negative parallel and η → π* negative transitions, respectively (Fig. 9A). CD of Hb adducted by either compound revealed a large change in the far UV region of the spectrum, consisting of a decrease in molar ellipticity at λ = 192 nm and an increase in molar ellipticity at λ = 208 and 222 nm indicate that the helicity was significantly decreased. The deconvoluted CD spectra and calculated compositions of secondary structure showed that the α-helix content for the human Hb control was 47.7%, which decreased to 24.4% in the presence of PhNHOH and to 21.1% in the presence of NOB (Supplementary Information Table S1).

Additionally, to determine if adducted Hb undergoes conformational changes induced by PhNHOH and NOB binding, UV-Vis absorption spectra were measured. Figure 9B shows the absorbance spectra between λ = 250–500 nm for the control and adducted human Hb. The adducted Hb exhibited an increase in the absorption region of λ = 280 nm. Adduction of Hb also resulted in a hypochromic red shift in the maximal absorbance from λ = 407 to 419 nm and from λ = 407 to 426 nm for PhNHOH and NOB, respectively.

Spectrophotometric changes in the visible region of Hb exposed to PhNHOH and NOB are shown in Fig. 9C. Both chemicals caused the disappearance of the typical double peaks of absorption at λ = 541 nm and λ = 578 nm associated with oxy-Hb, which were replaced by single maxima at λ = 543 and 556 nm for Hb adducted by PhNHOH and NOB, respectively. No additional peak at ~630 nm was noted, indicating minimal formation of met-Hb under the conditions used in this study.

## Discussion

In the present study, adduction with model peptides containing nucleophilic moieties (Cys, Lys, His) revealed that only free thiols were modified by the aniline metabolites PhNHOH and NOB, with the major products identified as sulfinamide and sulfonamide. These results were also consistent with quantum mechanical calculations at the DFT level. For both PhNHOH and NOB, the higher predicted reactivity index (ω^−^) determined for Cys (−1) as compared to the neutral forms of Cys, Lys, and His correlated with the selectivity of thiolate adduction observed with model peptides and proteins and the lack of detectable adduction of either Lys or His. Similar predictions of Cys thiolate reactivity with other electrophiles have been previously reported^13,32^. However, the presence of a conjugated π electron network in both electrophiles studied here suggests that they may be relatively weak electrophiles and thus would likely require higher concentrations in order to drive the second-order adduction reaction. The HSAB calculations also projected a higher reactivity for NOB compared to PhNHOH as the electrophile, a prediction borne out by our experimental findings. Although specific determination of rate constants was not performed in our study, previous studies have demonstrated the high correlation between HSAB calculated electrophilicity values (ω) and rate of adduction, particularly in the absence of significant steric impacts on rate^13^.

It has been reported that PhNHOH must be oxidized first to the nitroso form (NOB) for thiol adduction to occur, and it has been proposed that the iron in the heme group of Hb can facilitate this oxidation step^33,34^. However, our results with synthetic peptides also demonstrated that oxidation to NOB can occur spontaneously, a result that is in agreement with those previously reported by others. For example, Pathak *et al*. reported that conversion of the hydroxylamine derivative of aminobiphenyl to the nitroso form could occur via air oxidation^35^. In addition, Becker *et al*. found that under aerobic conditions PhNHOH can be degraded spontaneously in aqueous solution at physiological pH to form NOB, nitrobenzene, and azoxybenzene^36^. Additionally, we demonstrated that spontaneous oxidation of PhNHOH is inhibited under a reducing or an anaerobic environments, as no sulfinamide was formed under these conditions.

The adduction reaction with AA metabolites is complex, as other pathways might yield varying intermediates and products, particularly following *in vivo* exposure^37^. In addition, these pathways depend on specific thiol pKa and concentration. Accordingly, hemoglobin adduction at lower AA metabolite concentrations was also tested in the present study. There are essentially no data available on physiological levels of PhNHOH or NOB within the erythrocyte following human exposure to aniline, thus making direct comparison of our *in vitro* concentrations with those encountered *in vivo* problematic. However, like other published work examining *in vitro* modification of Hb thiols by reactive xenobiotics^38–40^, the present study was not designed to specifically model *in vivo* occupational or environmental exposure but instead to investigate mechanism of adduction. Nevertheless, considering the prolonged exposures often encountered in these settings (compared to the short incubation times employed here), and the heme-mediated oxidative cycling of PhNHOH and NOB demonstrated to occur within the erythrocyte to regenerate reactive species^16^, the findings of the present study may also have relevance to human exposures.

In this study, cysteine sulfinamides and sulfonamides were identified as the primary *in vitro* adduction products with PhNHOH and NOB in both human and bovine Hb. Lack of formation of the sulfonamide in a heme-less protein (β-LGB-A) exposed to NOB as here demonstrated suggests that the heme group facilitates the oxidation of sulfinamide to sulfonamide observed in Hb. Our results with anaerobic conditions and the reducing agent TCEP shed additional light on the products of thiol modification by aniline metabolites. These data confirm that an N-hydroxylated compound, such as PhNHOH, is first oxidized to the nitroso form (NOB), which then reacts with the free thiol to form the sulfinamide. The lack of adduction by PhNHOH in the presence of TCEP indicates that oxidation of the N-arylhydroxylamine to form the arylnitroso does not occur under these conditions. Alternatively, it is possible that PhNHOH is reduced to aniline by TCEP. It has been reported that hydroxylamines can oxidize tertiary phosphines^41^ and that, under certain conditions, TCEP could react with maleimide and haloacyl groups^42^. Further experimentation to assess whether PhNHOH can be reduced to aniline as a consequence of reducing agent treatment would therefore be valuable.

*In vitro* exposure studies with human whole blood demonstrated that sulfonamide products were not formed on Hb with either PhNHOH or NOB, despite the presence of heme. This important finding suggests that other blood components, including small thiols such as GSH, may be responsible for inhibiting further oxidation of the sulfinamide. It has been shown that NOB reacts with GSH to form PhNHOH, GSSG, and glutathione sulfinanilide^43^. In addition, other reaction pathways between nitrosoarenes and glutathione have been reported^44^. Accordingly, this phenomenon may underlie the absence of observable sulfonamide adducts of Hb in our whole blood experiments, a finding that could have important implications for *in vivo* exposure to aromatic amines.

It has been reported that arylsulfinamide adducts can be unstable under conditions of elevated temperature, in the presence of reducing agents, or with low pH, with the cysteine sulfinic acid as a major product^40,45^. However, our results show that sulfinamide adducts are relatively stable at physiological pH, as we observed sulfinamides in both intact proteins and proteolytic digests. This may be because we did not perform reduction prior to the tryptic digestion, since neither bovine nor human Hb have disulfide bonds.

We did observe the formation of low levels of sulfinic and sulfonic acid adducts in our tryptic peptide digests of both control and adducted protein. Although protein sulfinic acids may be hydrolysis products of sulfinamides, they are also products of free thiol reactions with reactive oxygen species (ROS)^46^. For example, tryptic peptides containing sulfinic and sulfonic acids on ^34^Cys of HSA were detected in samples of human plasma and serum^47^. The redox couple, PhNHOH and NOB, working as proton sources, can contribute to the accumulation of H_2_O_2_, which can react with free thiols to yield sulfenic, sulfinic, and sulfonic acids^33,48^. In addition to the *in vivo* oxidation of cysteines by ROS, *in vitro* oxidations have been reported during the reaction of Hb with an excess of NOB^38^. Finally, ROS-dependent thiyl free radical formation in rat Hb has also been reported with PhNHOH and NOB *in vitro* and *in vivo* ^49^. Further studies are required to determine the mechanism of formation of such products in intact protein and LysC/tryptic digests.

Our UV-Vis spectroscopy results demonstrated that Hb adducted by either compound undergoes structural changes. The observed increased absorption around 280 nm might be due to structural changes that expose aromatic residues, or by the covalent binding of the chemical compounds which adds an additional phenyl group. Hb exhibits intense absorption bands above λ = 320 nm. Strong absorption near λ = 400 nm (Soret band) is characteristic of hematoporphyrin proteins^50^. The porphyrin ring has been found to be highly flexible^51^. The oxidation state of the coordinated metal, oxygen binding, or structural changes near the heme group can distort the porphyrin group, affecting the Soret band^52,53^ and making it useful to distinguish Hb states^54^. The Soret band maxima for Hb-Fe(II)O_2_ (oxy-Hb), Hb-Fe(II) (deoxy-Hb), and Hb-Fe(III) (met-Hb) have been reported as λ = 415, 430, and 405 nm, respectively^55^. The hypochromic red shift in the Soret maxima observed following adduction by either PhNHOH or NOB observed in the present study is consistent with conversion primarily to deoxy-Hb. In the visible region, oxygenated Hb has two absorption maxima, at around λ = 542 nm and λλ = 578 nm; deoxygenated Hb exhibits only a single maximum at λ = 540–550 nm^54^. Both aniline metabolites caused loss of the double peaks and replacement with single maxima at λ = 543 and 556 nm for Hb adducted by PhNHOH for Hb adducted by NOB, respectively. These results are also consistent with conversion of oxygenated Hb to the deoxygenated form.

In contrast, the UV-Vis spectral data did not suggest further transformation of Hb to either met-Hb or degradative forms of Hb such as hemichrome. For example, no significant peak at ~630 nm, typical of met-Hb, was observed with either PhNHOH or NOB under the conditions used in this study. The formation of met-Hb is a prominent sign of aromatic amine overexposure in humans, resulting in symptoms of oxygen deprivation (cyanosis) leading to morbidity or even death^16,56^. The results of *in vitro* studies are less consistent, with some showing significant formation of met-Hb following aniline or metabolite treatment and others not. For example, Pathak *et al*. demonstrated a decrease in oxygenated Hb content and a time-dependent increase in met-Hb following *in vitro* exposure to PhNHOH^35^. In contrast, spectral changes consistent with deoxy-Hb formation were not reported. Differences in experimental conditions (*e.g*., amine:Hb molar ratios, incubation time, use of RBCs vs. purified Hb, presence or absence of reducing agents, etc.) may account for some or all of these inconsistencies. Finally, spectral changes indicative of hemichrome formation, *i.e*., loss of major peaks in the 500–600 nm range^57,58^, were not noted in the present study, further suggesting that Hb oxidation did not proceed beyond the deoxy-Hb stage.

There are no previous published reports of changes in secondary structure of Hb assessed by CD following exposure to either PhNHOH or NOB. In the present study, the α-helical content of Hb was reduced by 49% with PhNHOH and by 56% with NOB, indicative of a strong disruption of native conformation of the protein. These results are consistent with those previously reported following treatment of Hb with other exogenous compounds. For example, far-UV CD spectral changes indicating a loss of secondary structure in Hb induced by the surfactant tetradecyltrimethylammonium bromide were attributed to increased hydrophobic interactions and subsequent loss of heme^59^. In another recent study, CD spectroscopy revealed loss of α-helicity in Hb with the fluorinated hydrocarbon perfluorooctane sulfonate (PFOS), linked to changes in heme group symmetry following entry of PFOS into the binding cavity of Hb^60^. When coupled to the UV-Vis results, our CD data suggest that, following thiol adduction, Hb may not be able to function as an oxygen carrier due to changes in secondary structure that are the direct result of covalent modification of free cysteines in Hb.

In view of the proximity of β^93^Cys to the heme moiety, it would not be unexpected that its modification would lead to important changes within the microenvironment of this residue. β^93^Cys is conserved among vertebrates and is a functionally significant residue of human Hb, and adduction of β^93^Cys by aromatic amines has been recognized for decades^23,28,35,38,61,62^. However, in addition to modification of β^93^Cys of human Hb, we found two novel sites of adduction; α^104^Cys and β^112^Cys. Unlike β^93^Cys in human Hb, these residues are buried at the α_1_β_1_ interface in Hb and generally less accessible to attack by potential electrophile^63^. However, α^104^Cys and β^112^Cys are likely to be involved in the quaternary structure of Hb^64^, with β^112^Cys identified as a key amino acid in stabilization of the αβ dimer by hydrophobic interactions with the α subunit^65^. Additionally, this interface is involved in structural changes of Hb that affect oxygen affinity. Analysis of the reactivity of the free thiols in the oxy and deoxy-Hb states reveal that β^93^Cys is generally reactive, while β^112^Cys is reactive in deoxy-Hb but unreactive in oxy-Hb^66^.

Kan *et al*. recently utilized the sulfhydryl reagent, *p*-hydroxymercuribenzoate (PMB) to prove that all three cysteine residues, the surface-exposed β^93^Cys and the shielded α^104^Cys and β^112^Cys, could be modified^39^. These authors reported that PMB-modification proceeded in a stepwise manner, with a reactivity order of: β^93^Cys > α^104^Cys > β^112^Cys. The modification of these residues will prompt a complete quaternary structure disassembly due to a disruption of the intersubunits within salt bridge networks. Based on these findings, adduction of all three cysteines by PhNHOH and NOB, as demonstrated in the present study, would be expected to induce conformational changes to induce the deoxy-Hb form and to destabilize the quaternary Hb structure. Comparable results have been observed in Hb adducted with methyl bromide, where all three cysteines were methylated^67^.

In summary, we utilized the aniline metabolites PhNHOH and NOB as model reactive xenobiotics in an *in vitro* peptide/protein modification system and in *in silico* reactivity calculations for a better understanding of reaction products and nucleophilic targets. Our studies reveal that, as predicted by *in silico* modeling, protein Cys thiol moieties, and not His or Lys residues, are the preferred reaction sites for both metabolites. In addition, we evaluated reaction conditions (*i.e*., protein:metabolite molar ratio, exposure time and pH, presence or absence of reducing agents and O_2_) impacting the modification of free thiol moieties into sulfinamides and/or sulfonamides in purified human Hb exposed to these agents. Results of our reaction site studies are in general agreement with previous reports demonstrating selective modification of β^93^Cys in human hemoglobin^23,28,35,38,61,62^. However, we also provide new data on modification of the two other free thiols in human Hb, β^112^Cys and α^104^Cys. Our data indicate that sulfinamide adducts are stable in intact protein and during proteolytic digestion, a finding that facilitates further proteomic analyses of these reactions. Finally, data consistent with changes in Hb oxidation state, in addition to structural alterations as a result of Hb thiol adduction, are also reported.

## Methods

***Caution:*** *PhNHOH and NOB are toxicants and potential carcinogens that should be handled in fume hoods with appropriate personal protective equipment*.

### Chemicals and Materials

PhNHOH, NOB, bovine Hb, human Hb, β-lactoglobulin A from bovine milk (β-LGB-A), iodoacetamide (IAM), DTT, GSH, GSSG, and sequencing grade trifluoroacetic acid (TFA) were obtained from Sigma-Aldrich (St. Louis, MO). LysC/trypsin mix was obtained from Promega Corporation (Madison, WI). The model synthetic peptides AcPAAKAA, AcPAACAA and AcPAAHAA were custom synthesized by Biomatik, Inc. (Wilmington, DE). Human acetyl ACTH (1–17) and acetyl γ-endorphin peptides were obtained from American Peptide Company (Vista, CA). ELHCDKL peptide was synthesized in-house using Fmoc-Leu-Wang resin with amino acids obtained from AnaSpec Inc. (San Jose, CA). Amicon Ultra Centrifugal Filters, 0.5 mL/10 kDa and C18 Zip-Tips were from Millipore Ltd. (Bedford, MA). Pooled human whole blood from random, deidentified donors was obtained from a commercial supplier (Bioreclamation, LLC; Westbury, NY). All solvents were high purity grade from Fisher Scientific Co. (Pittsburgh, PA).

### Liquid Chromatography Mass Spectroscopy

LC-MS and MS/MS peptide analyses were performed on an Agilent 1290 Infinity UHPLC coupled to an Agilent model 6460 triple quadrupole-MS (LC-QqQ-MS) equipped with JetStream ESI. Mobile phase A consisted of 0.1% TFA in HPLC grade water and mobile phase B 95:5:0.1% acetonitrile/water/TFA. Peptides were analyzed by direct flow injection with an isocratic mobile phase of 85% A and 15% B at a flow of 0.5 mL/min. Analyses were conducted in positive ionization mode. MS/MS was conducted on the singly charged state of the precursor ion. Data acquisition analyses were processed using Agilent MassHunter software package (Version B.0.6.0).

Whole protein MS was performed using an Agilent 1290 Infinity UHPLC coupled to an Agilent 6530 quadrupole time-of-flight (LC-QTOF) mass spectrometer with a mass resolution (resolving power) of 20,000 for smaller proteins (<30 kDa), which allowed for the detection of peptide and protein adducts. Direct flow injections of whole protein solutions into the ESI were performed using an isocratic mobile phase of 50% A and 50% B at a flow of 0.3 mL/min. The spectra were deconvoluted using a Maximum Entropy charge algorithm contained within the Agilent MassHunter BioConfirm software (Version B.0.6.0).

Protein digests were analyzed with an Orbitrap Fusion™ Lumos™ Tribrid™ Mass Spectrometer (Thermo Fisher Scientific) operated in positive mode, with a mass resolution (resolving power) of 1,000,000 FWHM. LC-MS/MS was operated in DDA mode, with an MS/MS cycle time of 3 s. Precursors were sorted by charge state with selected charges of +1, +2 and +3. Peptide separations were carried out on a Thermo Scientific C18 RP-HPLC column (2 μm, 75 μm i.d. × 250 mm). A 75 min gradient of 95% mobile phase A (water, 0.1% formic acid) 5% to 27.5% mobile phase B (80% acetonitrile, 0.08% formic acid) over 43 min followed by a ramp to 40% mobile phase B over 7 min and lastly to 95% mobile phase B over 5 min at a flow rate of 300 nL/min was used to separate the peptides. Data analysis was carried out using Proteome Discoverer software (Thermo Fisher Scientific; Version 2.0). The chemical modifications included in the analysis were sulfenic, sulfinic, and sulfonic acid, sulfinamide, sulfonamide, sodium and potassium adducts, alkylation by iodoacetamide, and heme group. Protein sequences were obtained from UniProt^68^.

### Peptide Synthesis

ELHCDKL model peptide was synthesized on a solid phase using Fmoc chemistry^69^ starting from Fmoc-Leu-Wang resin using a PS3™ (Protein Technologies Inc.) synthesizer. The peptide was cleaved from the resin with 94% TFA, 2.5% H_2_O, 2.5% 1,2-ethanedithiol, and 1% thioanisole. Peptide purifications were performed by RP-HPLC on a C18 semipreparative column (Vydac, 218TP510, 10 mm 250 mm; 5 μm particle diameter; 300 Å pore size) with a C18 guard column (Upchurch Scientific, AC-43 4.6 mm) at a flow rate of 3.5 mL/min. For the RP-HPLC separation, mobile phase A was 0.1% TFA and mobile phase B was 0.1% TFA in 60% acetonitrile. The peptide was eluted with an incremental linear gradient of 1% solvent B/min with monitoring at λ = 220 nm. The correct peptide mass and sequence were confirmed by LC-QqQ-MS/MS.

### HSAB Quantum Mechanical Calculations

Density Functional Theory (DFT) calculations to approximate the properties of chemical potential (μ), chemical hardness (η), chemical softness (σ), electrophilicity index (ω), and reactivity index (ω^−^) were performed by determining *E*_HOMO_ and *E*_LUMO_ for reactive electrophiles (PhNHOH and NOB) and nucleophilic target sites (Cys, His, and Lys) as previously described^9,32^. Briefly, initial structure geometries were generated via molecular orbital package (MOPAC) optimization using Chem3D Ultra software (Version 8, CambridgeSoft Corporation, Waltham, MA) and were exported to Gaussian 03 software (Gaussian, Inc., Wallingford, CT). Iterative structural geometry optimization was carried out, and *E*_HOMO_ and *E*_LUMO_ were calculated at the density functional level using a B3LYP functional with 6–31 G* basis set within Gaussian 03. Existing literature defines ω^−^ as “nucleophilicity index”; however, for this work, ω^−^ was termed “reactivity index” to indicate its potential to predict adduct formation between a specified nucleophile and electrophile.

### Preparation of PhNHOH and NOB peptide adducts

Peptides (0.1 mg/mL) were dissolved in 25 mM ammonium bicarbonate buffer (pH 7.8) and treated with a molar excess of PhNHOH or NOB to peptide ranging up to 30:1. Samples (triplicate) were incubated in the dark for 3 h at 25 °C. Additionally, in some experiments peptides were alkylated at Cys residues prior to adduction by incubation for 1 h with 6 mM IAM, in order to confirm that adduction did not take place when the free thiol was blocked. In other experiments, peptides were adducted first and then subjected to reaction with IAM to assess stability of the adduct under these conditions. Peptide masses were confirmed using LC-QqQ-MS. Controls consisted of peptides incubated in parallel assays without the addition of PhNHOH or NOB. Anaerobic incubations were performed by initially purging all solutions with argon for 30 min before use. Peptides were dissolved in purged ammonium bicarbonate buffer and then purged again for 20 min. Selected aniline metabolites were added to peptide solutions while the sample mix was continuously bubbled with argon, incubated for 1 h at 25 °C, and then immediately analyzed by LC-QqQ-MS/MS.

### Modification of proteins with PhNHOH and NOB

Protein adduction reactions were carried out as described previously^38^ with slight modifications. Briefly, protein solutions (2.5 mg/mL) in 25 mM ammonium bicarbonate buffer (pH 7.8) were incubated with PhNHOH or NOB. The proteins used were β-LGB-A, bovine Hb, and human Hb. β-LGB-A and bovine Hb were included to simplify initial MS studies, as they only contain a single reactive thiol site. In addition, β-LGB-A does not contain a heme group, thus allowing examination of the possible effect of heme on adduction by comparison to Hb. Molar excesses of PhNHOH or NOB to protein ranging from 0.005- to 30-fold (corresponding to 0.75 μM to 4.5 mM concentration) were examined. PhNHOH and NOB were dissolved in acetonitrile prior to use. Samples prepared in triplicate were incubated in the dark overnight at 25 °C with gentle agitation. Controls consisted of proteins incubated in parallel assays without the addition of PhNHOH or NOB. Unreacted chemicals were removed from the mixtures by centrifugal filtration with three washes using equal volumes of buffer. Samples were analyzed by direct flow injection and spectral deconvolution on the LC-QTOF-MS.

### Proteolytic digestion

Human and bovine Hb control and adducted proteins were alkylated with 15 mM IAM for 1 h. Centrifugal filters were used to eliminate excess alkylating agent. Proteins were resuspended in 25 mM ammonium bicarbonate buffer (pH 7.8) containing 1 mM of CaCl_2_. Then, proteins were digested with LysC/trypsin at an enzyme:protein ratio of 1:200 (w/w) at 37 °C for 18 h. Tryptic peptides were concentrated with C18 Zip-Tips. Proteolytic peptides were separated and analyzed using an Orbitrap Fusion Lumos Tribrid MS/MS. Spectra were analyzed with Proteome Discoverer™ Software (Version 2.0).

### *In vitro* whole blood exposure to PhNHOH and NOB

Heparinized pooled human blood samples (1 mL) from a commercial source were exposed to 1 mM of PhNHOH or NOB. The pH of the intact whole blood at room temperature was 7.6. Samples were incubated in the dark overnight at 25 °C with gentle agitation. Hb purification was carried out as described previously^70^ with slight modifications. Briefly, RBCs were separated by centrifugation at 1,000 × *g* at 4°C for 30 min. The pellet was recovered and washed three times with equal volumes of 310 mM Tris buffer (pH 7.6). Washed RBCs were then lysed via forceful syringe injection of six volumes of 20 mM Tris buffer (pH 6.7). Lysed cells were centrifuged at 20,000 × *g* for 40 min at 4 °C. The hemolysates were added drop-wise to 20 volumes of cold (−20°C) acetone. Samples were centrifuged at 1000 × *g* for 10 min and pellets were dried under N_2_ and reconstituted in 25 mM ammonium bicarbonate buffer (pH 7.8). Samples were analyzed by direct flow injection on the LC-QTOF-MS.

### Human Hb adduction in presence of GSH/GSSG

Commercial human Hb was dissolved in 25 mM ammonium bicarbonate buffer (2.5 mg/mL; pH 7.8) and incubated with 0.4 mg/mL of GSH, 0.05 mg/mL of GSSH, and a 30-fold molar excess of PhNHOH or NOB (4.5 mM final concentration). The GSH:GSSG ratio used was 8:1, in accordance with previous studies^31^. Samples were incubated in the dark overnight at 25 °C with agitation and then filtered. Samples were analyzed by direct flow injection on the LC-QTOF-MS.

### Circular dichroism spectropolarimetry

A stock solution of 7 mg/mL of human Hb was prepared and divided into three aliquots of 500 μL each: one control and two treated with a three-fold molar excess of PhNHOH and NOB (1.3 mM final concentration), respectively, for 16 h. Unreacted chemicals were removed from the mixtures by use of centrifugal filters. Human Hb samples were diluted to 0.25 mg/mL in 25 mM ammonium bicarbonate (pH 7.8). Far-UV CD spectra were measured on a Jasco-810 spectropolarimeter (Easton, MD) using a quartz cell of 1 mm optical path length. Measurements were carried out between λ = 190–260 nm at 100 nm/min with a response time of 1 s. The CD spectrum obtained was the result of an average of 16 scans recorded at 25 °C. All CD spectra were background-corrected for the buffer signal. The reported secondary structure percentage values are the average results computed by CDNN^71^, CDSSTR^72^, SELCON 3^73,74^, and CONTINLL^75^ software.

### UV-Visible spectroscopy

UV-Visible absorption spectra were measured with a Genesys 10 S UV-Vis spectrophotometer (Thermo Scientific; Madison, WI). Samples were prepared as described above. Human Hb concentrations were 0.25 mg/mL for UV-visible absorption (250–500 nm) and 2 mg/mL for visible absorption (450–650 nm) with 1 nm increments.

### Data availability

Most data generated or analyzed during this study are included in this published article (and its Supplementary Information file). Data not reported are available from the corresponding author on reasonable request.

### Disclaimer

Certain commercial equipment, instruments, or materials are identified in this paper in order to specify the experimental procedure adequately. Such identification is not intended to imply recommendation or endorsement by the National Institute of Standards and Technology, nor is it intended to imply that the materials or equipment identified are necessarily the best available for the purpose.

## Electronic supplementary material

Supplementary Information
