# Supplementary material for: Proteomic Analysis of Thiol Modifications and Assessment of Structural Changes in Hemoglobin Induced by the Aniline Metabolites N-Phenylhydroxylamine and Nitrosobenzene
Source: Sci Rep. 2017 Nov 1;7:14794. doi: 10.1038/s41598-017-14653-w (PMC5665987; doi:10.1038/s41598-017-14653-w)
Supplement: Supplementary file 1 — Supplementary Information [file 41598_2017_14653_MOESM1_ESM.pdf]

SUPPLEMENTARY INFORMATION

Proteomic Analysis of Thiol Modifications and Assessment of Structural Changes in Hemoglobin Induced by Aniline Metabolites N-Phenylhydroxylamine and Nitrosobenzene

Carolina Möller[1,2], W. Clay Davis[2], Vanessa R. Thompson[1], Frank Marí[2] and Anthony P. DeCaprio[1]

[1]Department of Chemistry and Biochemistry, Florida International University, 11200 SW 8th St, Miami, FL 33199, USA.

[2]Marine Biochemical Sciences, Hollings Marine Laboratory, National Institute of Standards and Technology, 331 Fort Johnson Rd., Charleston, SC 29412, USA.

Correspondence and requests for materials should be addressed to APD (email: adecapr@fiu.edu).

Table S1. Human hemoglobin secondary structure estimation from CD spectra by different software.

Sample	Software	Helix	Antiparallel	Parallel	Beta-Turn	Random Coil
**H-Hb Control**	CDNN	44.4%	2.3%	7.5%	14.4%	31.8%
	CDSSTR	49.1%	5.2%	7.9%	14.4%	23.0%
	SELCON3	46.9%	5.2%	7.3%	15.5%	24.5%
	CONTINLL	50.3%	4.8%	4.9%	14.8%	25.2%
	Average	47.7%	4.4%	6.9%	14.8%	26.1%
**H-Hb + PhNHOH**	CDNN	27.6%	12.8%	10.2%	17.8%	38.4%
	CDSSTR	22.0%	9.2%	17.2%	21.5%	29.5%
	SELCON3	20.9%	9.7%	17.7%	23.0%	28.5%
	CONTINLL	27.0%	8.4%	14.0%	20.3%	30.2%
	Average	24.4%	10.0%	14.8%	20.7%	31.7%
**H-Hb + NOB**	CDNN	25.0%	17.7%	10.7%	18.5%	38.9%
	CDSSTR	18.2%	10.1%	18.5%	21.9%	30.7%
	SELCON3	18.0%	9.6%	18.7%	18.2%	22.9%
	CONTINLL	23.1%	10.0%	16.4%	20.8%	29.7%
	Average	21.1%	11.9%	16.1%	19.8%	30.5%

Figure S1

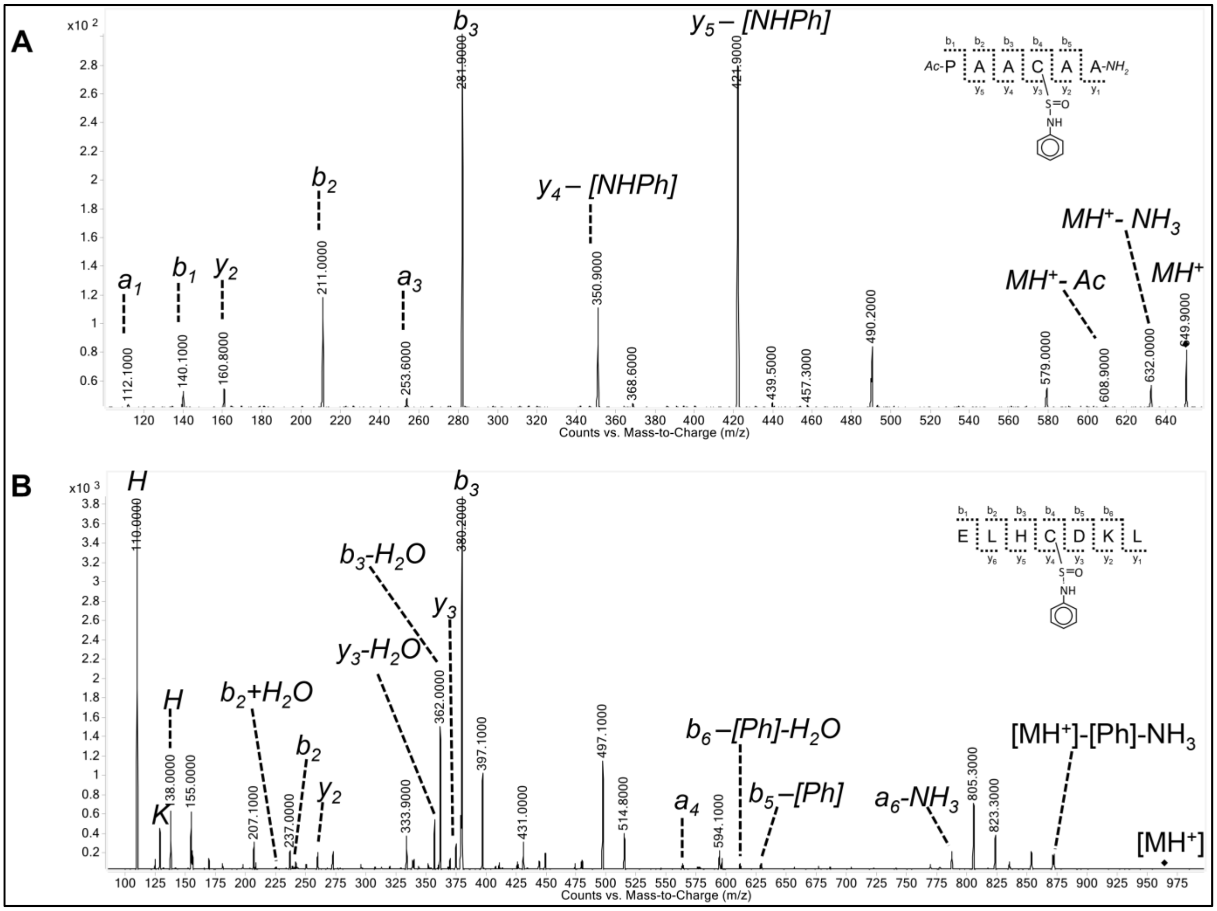

Figure S1: LC-ESI-QqQ-MS/MS product ion spectra of sulfinamide adducted peptides. MS/MS conducted on singly charged precursor ions $[M+H]^+$. A) Product ion spectrum of the Cys sulfinamide adducted peptide Ac-PAAGCAA-NH_2. B) Product ion spectrum of the Cys sulfinamide adducted β-Hb moiety peptide ELHCDKL. Assigned ions are represented in main text Table 3.

Figure S2

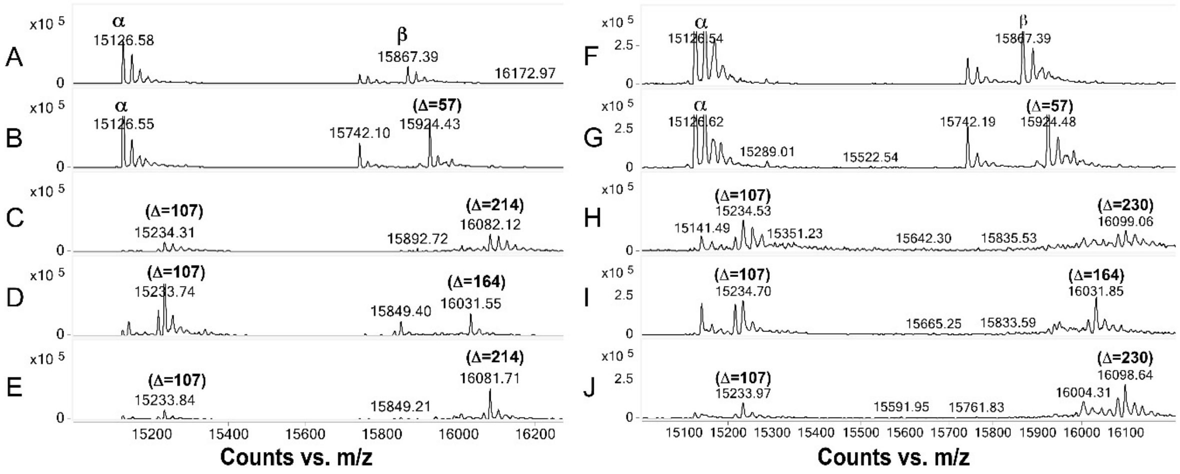

Figure S2: Adduction of Human Hb in presence of IAM. Left panels correspond to adduction with PhNHOH and right panels with NOB. A) Human Hb control, B) Human Hb incubated with IAM, showing an increase of 57 Da corresponding to the alkylation of one cysteine on β-subunit, C) Human Hb incubated with PhNHOH, showing increases of 107 and 214 Da, corresponding to formation of one and two sulfinamides on the α-subunit and the β-subunit, respectively, D) Treatment of human Hb with PhNHOH after incubation with IAM. A sulfinamide adduct (+107 Da) is present on the α-subunit, while a combination of one alkylated cysteine plus a sulfinamide (+164 Da) is present on the β-subunit. E) Human Hb treated first with PhNHOH and then incubated with IAM. A sulfinamide adduct (+107 Da) is present on the α-subunit, while two sulfinamides (+214 Da) are present on the β-subunit. No displacement of adducts by IAM was observed. F) Human Hb control, G) Human Hb incubated with IAM, showing an increase of 57 Da corresponding to the alkylation of one cysteine on the β-subunit, H) Human Hb incubated with NOB, showing increases of 107 Da and 230 Da corresponding to formation of one sulfinamide and a combination of one sulfinamide plus one sulfonamide on the α-subunit and the β-subunits, respectively, I) Treatment of human Hb with NOB after incubation with IAM. A sulfinamide adduct (+107 Da) is present on the α-subunit, while a combination of one alkylated cysteine plus a sulfinamide (+164 Da) is present on the β-subunit. J) Human Hb treated first with NOB and then incubated with IAM. A sulfinamide adduct (+107 Da) is present on the α-subunit, while a combination of one sulfinamide and one sulfonamide (+230 Da) is present on the β-subunit. No displacement of adducts by IAM was observed.

Figure S3

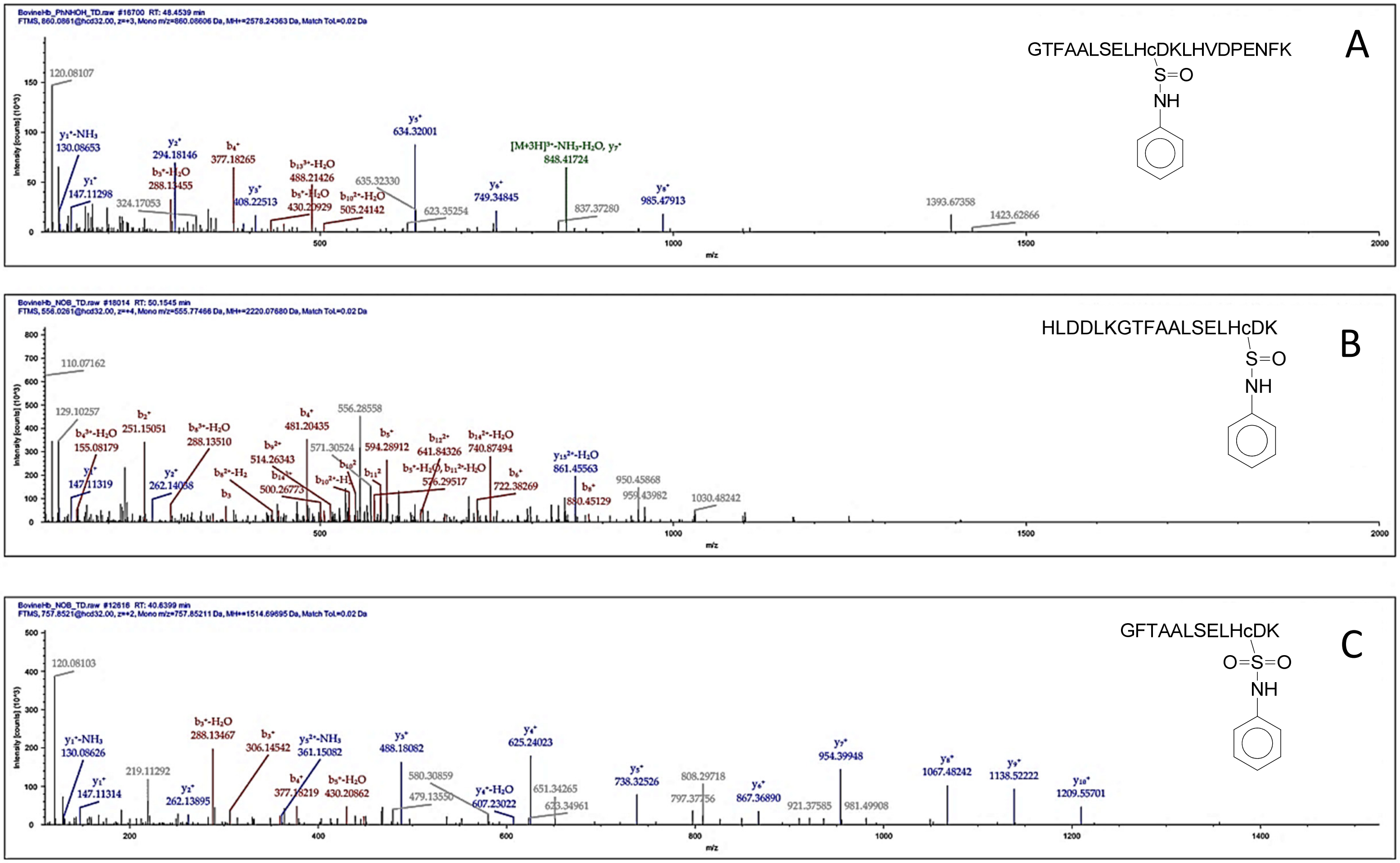

Figure S3: LC- MS/MS product ion spectra of the digested peptides of adducted bovine β-Hb, along with deduced structure. A) Product ion spectra of sulfinamide on ^{91}Cys of the partial digested peptide between residues 80 and 102, precursor ion $[M+3H]^{3+}$. B) Product ion spectra of sulfinamide on ^{91}Cys of the partial digested peptide between residues 74 and 93, precursor ion $[M+4H]^{4+}$. B) Product ion spectra of sulfonamide on 91Cys of the digested peptide between residues 81 and 93, precursor ion $[M+2H]^{2+}$. Assigned ions are represented in main text Table 4. Ions in blue are y-series, ions in red are b-series, ions in green are other peptide ions, ions in gray are unassigned.

Figure S4

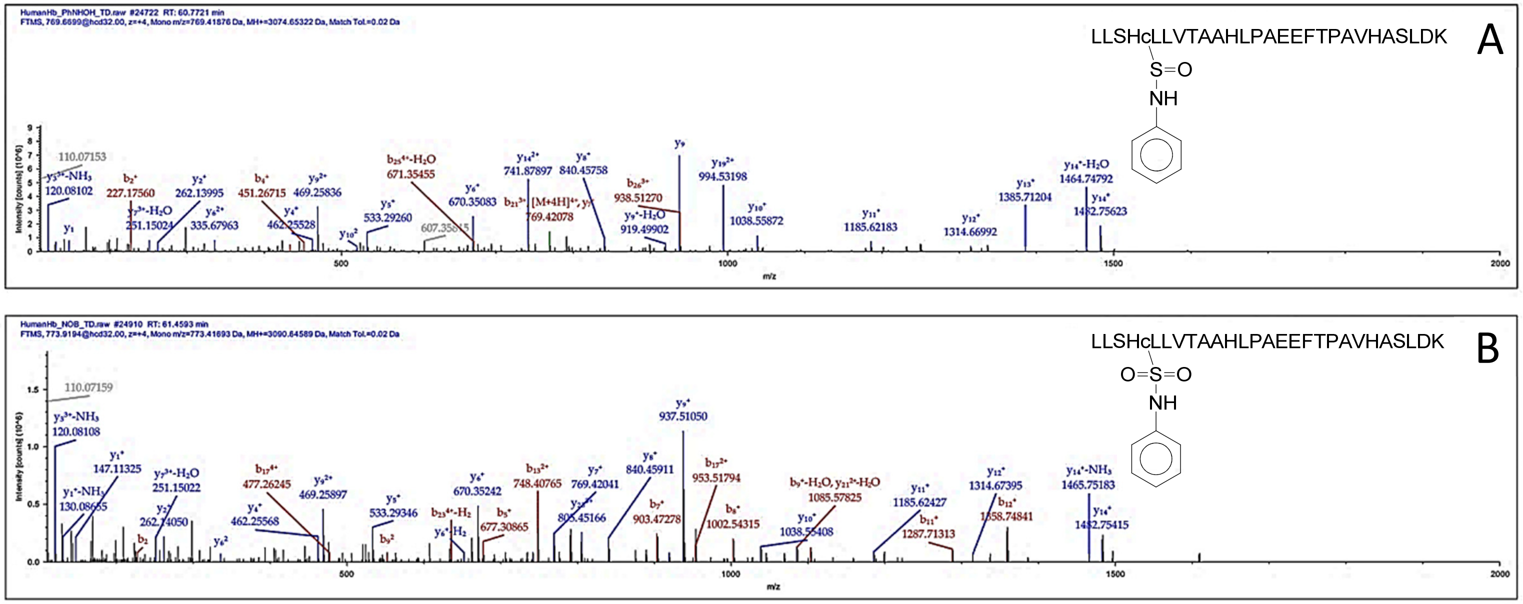

Figure S4: LC -MS/MS product ion spectra of the digested peptides of adducted human α-Hb, along with deduced structure. A) Product ion spectrum of sulfinamide on [104]Cys of human α-Hb of the digested peptide between residues 100 and 127, precursor ion $[M+4H]^{4+}$. B) Product ion spectrum of sulfonamide on [104]Cys of human α-Hb of the digested peptide between residues 100 and 127, precursor ion $[M+4H]^{4+}$. Assigned ions are represented in main text Table 4. Ions in blue are y-series, ions in red are b-series, ions in gray are unassigned.

Figure S5

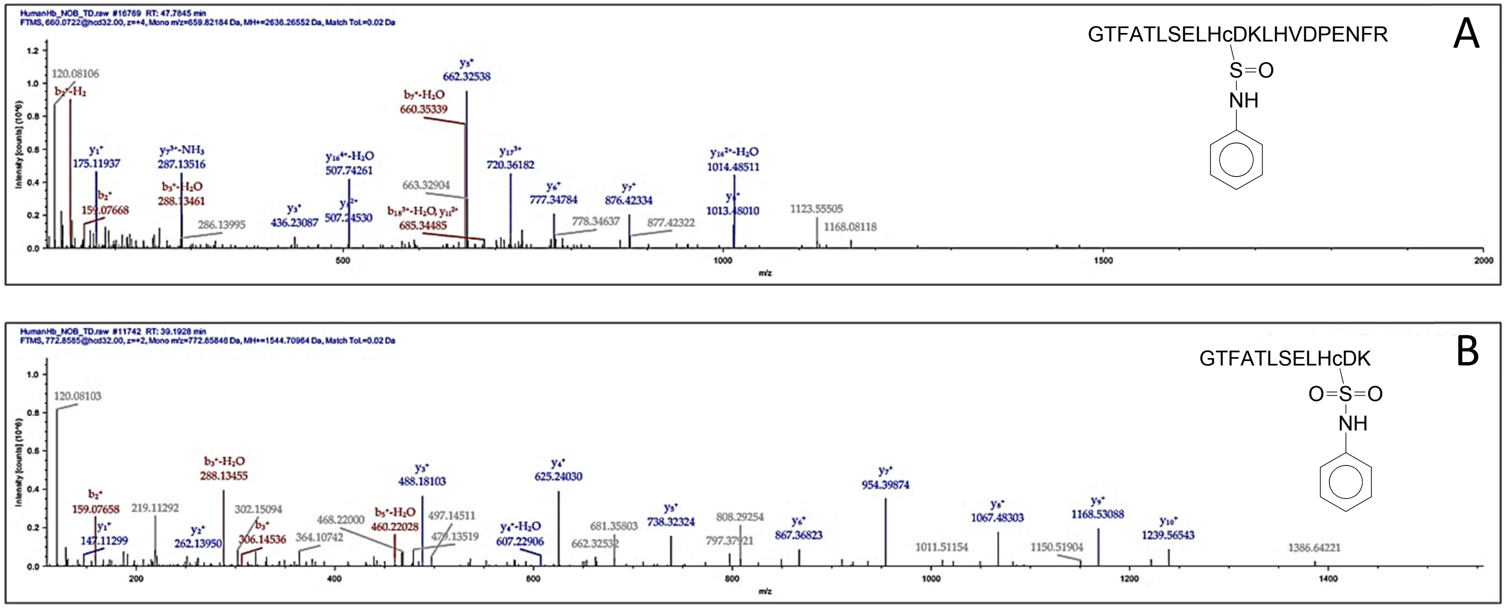

Figure S5: LC-MS/MS product ion spectra of the digested peptides of adducted human β-Hb, along with deduced structure. A) Product ion spectrum of sulfinamide on [93]Cys-93 of human β-Hb of the digested peptide between residues 83 and 104, precursor ion $[M+4H]^{4+}$. B) Product ion spectrum of sulfonamide on [93]Cys of human α-Hb of the digested peptide between residues 83 and 95, precursor ion $[M+2H]^{2+}$. Assigned ions are represented in main text Table 4. Ions in blue are y-series, ions in red are b-series, ions in gray are unassigned.

Figure S6

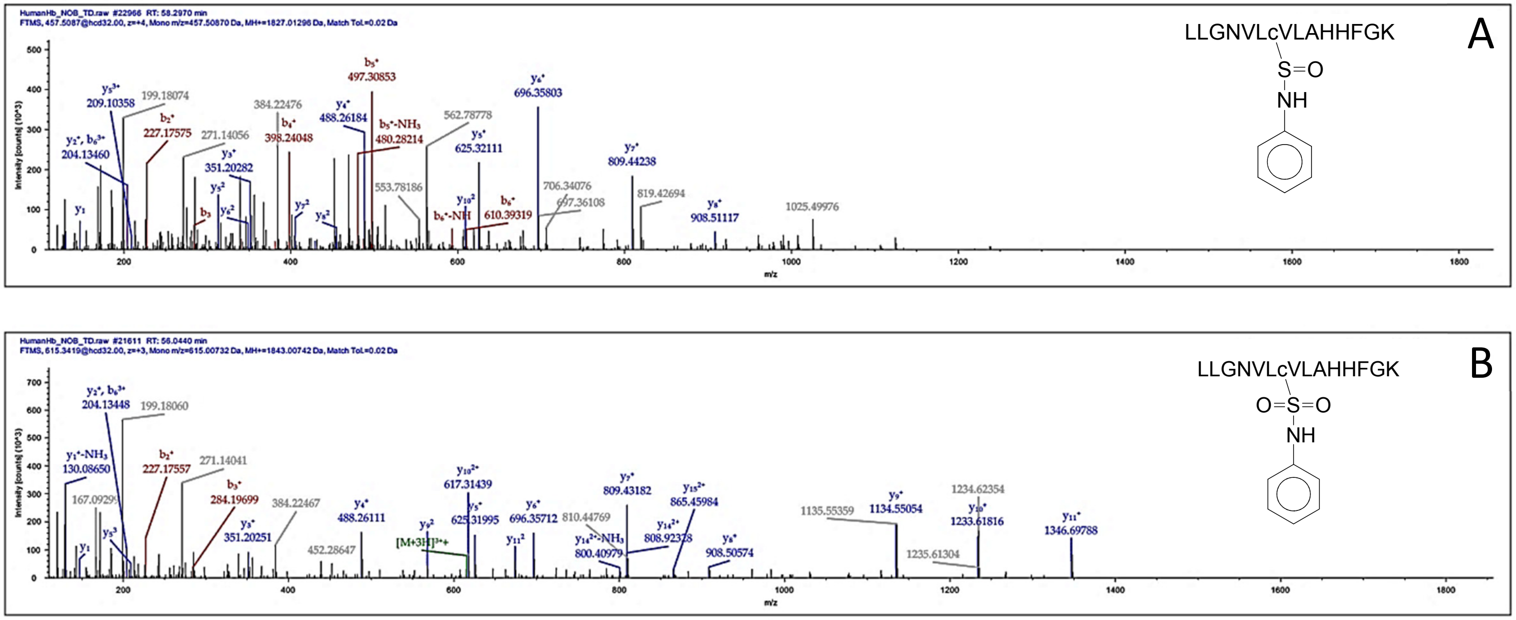

Figure S6: LC-MS/MS product ion spectra of the digested peptides of human β-Hb, along with deduced structure. A) Product ion spectrum of sulfinamide on [112]Cys of human β-Hb of the digested peptide between residues 105 and 120, precursor ion $[M+4H]^{4+}$. B) Product ion spectrum of sulfonamide on [112]Cys of human β-Hb of the digested peptide between residues 105 and 120, precursor ion $[M+3H]^{3+}$. Assigned ions are represented in main text Table 4. Ions in blue are y-series, ions in red are b-series, ions in green are other peptide ions, ions in gray are unassigned.
